# Evolutionary trajectory of fish *Piscine novirhabdovirus* (=Viral Hemorrhagic Septicemia Virus) across its Laurentian Great Lakes history: Spatial and temporal diversification

**DOI:** 10.1002/ece3.6611

**Published:** 2020-09-02

**Authors:** Carol A. Stepien, Megan D. Niner

**Affiliations:** ^1^ Genetics and Genomics Group (G3) NOAA Pacific Marine Environmental Laboratory (PMEL) Seattle WA USA; ^2^ Genetics and Genomics Group (G3), Department of Environmental Sciences University of Toledo Toledo OH USA

**Keywords:** evolutionary diversification, fish disease, novirhabdovirus, quasispecies, rhabdovirus, RNA virus, Viral Hemorrhagic Septicemia Virus

## Abstract

*Piscine novirhabdovirus* = Viral Hemorrhagic Septicemia Virus (VHSV) first appeared in the Laurentian Great Lakes with large outbreaks from 2005 to 2006, as a new and novel RNA rhabdovirus subgenogroup (IVb) that killed >30 fish species. Interlude periods punctuated smaller more localized outbreaks in 2007, 2010, and 2017, although some fishes tested positive in the intervals. There have not been reports of outbreaks or positives from 2018, 2019, or 2020. Here, we employ a combined population genetics and phylogenetic approach to evaluate spatial and temporal evolutionary trajectory on its *G‐*gene sequence variation, in comparison with whole‐genome sequences (11,083 bp) from a subset of 44 individual isolates (including 40 newly sequenced ones). Our results show that IVb (*N* = 184 individual fish isolates) diversified into 36 *G*‐gene haplotypes from 2003 to 2017, stemming from two originals (“a” and “b”). *G*‐gene haplotypes “a” and “b” differed by just one synonymous single‐nucleotide polymorphism (SNP) substitution, remained the most abundant until 2011, then disappeared. Group “a” descendants (14 haplotypes) remained most prevalent in the Upper and Central Great Lakes, with eight (51%) having nonsynonymous substitutions. Group “b” descendants primarily have occurred in the Lower Great Lakes, including 22 haplotypes, of which 15 (68%) contained nonsynonymous changes. Evolutionary patterns of the whole‐genome sequences (which had 34 haplotypes among 44 isolates) appear congruent with those from the *G*‐gene. Virus populations significantly diverged among the Upper, Central, and Lower Great Lakes, diversifying over time. Spatial divergence was apparent in the overall patterns of nucleotide substitutions, while amino acid changes increased temporally. VHSV‐IVb thus significantly differentiated across its less than two decades in the Great Lakes, accompanied by declining outbreaks and virulence. Continuing diversification likely allowed the virus to persist at low levels in resident fish populations, and may facilitate its potential for further and future spread to new habitats and nonacclimated hosts.

## Graphical abstract

This study analyses the evolutionary trajectory of a novel virus over its 17‐year history, examining the dynamics of its host relationships and its patterns across geographic space and evolutionary time. We sequence its entire genome and discern that the virus has significantly changed, with its original genotypes disappearing and being replaced in a quasispecies trajectory. It also has become less virulent, appearing to adapt for long‐term persistence at low levels in the host populations.

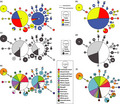

## 1 INTRODUCTION

Coevolutionary responses of viruses and their host populations offer intriguing insights into the myriad of genetic pathways that may ensue over time and space. RNA viruses possess rapid evolutionary rates due to their small genomes, lack of mutation proofreading, and short generation times (Holmes, 2009; Sanjuán, Nebot, Chirico, Mansky, & Belshaw, 2010; Volz, Koelle, & Bedford, 2013). Some rapidly diversify into multidirectional “cloud‐like” bursts of closely related variants, stemming from one or more central ancestral types, following a “quasispecies” evolutionary pattern (Andino & Domingo, 2015; Belshaw, Gardner, Rambaut, & Pybus, 2008). The pool of variants may serve as a genetic reservoir, facilitating adaptation to new hosts and environments (Andino & Domingo, 2015; Lauring & Andino, 2010; Quer et al., 1996). Quasispecies diversification patterns have been described for Avian Leukosis Virus (Meng et al., 2016), Deformed Wing Virus (Mordecai, Wilfert, Martin, Jones, & Schroeder, 2015), and *Piscine novirhabdovirus* (=Viral Hemorrhagic Septicemia Virus [VHSV]) across its world range (Pierce & Stepien, 2012; Stepien, Leaman, & Niner, 2020; Stepien, Pierce, Leaman, Niner, & Shepherd, 2015). The present study analyzes the latter's endemic IVb subgenogroup across its <20‐year history in the Laurentian Great Lakes, employing a combined phylogenetic and population genetics approach to aid understanding of virus–host evolutionary dynamics.

Phylogenetic approaches have elucidated the overall evolutionary patterns of emerging and resurging viruses, such as Zika (Faye et al., 2014), West Nile (May, Li, Davis, Galbraith, & Barrett, 2010), Measles (Kimura et al., 2015), and the VHSV fish virus (Pierce & Stepien, 2012; Stepien et al., 2015, 2020). Such broadscale examinations generally have lacked the fine‐scale resolution to address recent temporal and spatial trends, which can be assessed with a population genetics approach. Population genetic investigations analyze changes in gene frequencies over spatial and temporal scales to discern the effects of natural selection, drift, and gene flow on mutational variation, as well as their respective influences on fitness and adaptations (summarized by Hedrick, 2011; Lowe, Kovach, & Allendorf, 2017).

Most traditional virus studies have defined a viral population as a genetic isolate from a single host individual (Beerenwinkel & Zagordi, 2011; Yang et al., 2012). Here, we define a viral population as the gene pool obtained from isolates at a given geographic location and from a common time point, comprising a single outbreak. The few population genetic studies of viruses to date largely have been restricted to agriculturally important plant viruses (Alabi, Martin, & Naidu, 2010; Lin, Rubio, Smythe, & Falk, 2004; Tsompana, Abad, Purugganan, & Moyer, 2005) or human pathogens (Bahl, Vijaykrishna, Holmes, Smith, & Guan, 2009; Kearney et al., 2009; Pesko & Ebel, 2012). Most examined viral populations either within a single host species (Alabi et al., 2010; Pesko & Ebel, 2012; Tsompana et al., 2005) or a single host individual (Kearney et al., 2009), rather than focusing on broadscale evolutionary trends across a virus' temporal and spatial history, as addressed here.

**FIGURE 1:**
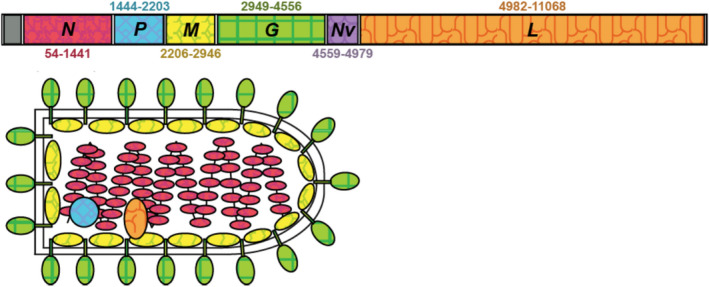
VHSV‐IVb structure and genome. Colors match the gene to the structure diagram. Numbers sharing the same colors as the gene refer to the nucleotide positions. Modified with permission from Pore (2012)

*Piscine novirhabovirus* (VHSV) is a negative‐sense RNA rhabdovirus of 11,158 nucleotides (NTs) that encode six genes (Figure 1): nucleoprotein (*N*), phosphoprotein (*P*), matrix protein (*M*), glycoprotein (*G*), nonvirion (*Nv*), and large protein (*L*), as 5′‐*N*‐*P*‐*M*‐*G*‐*Nv*‐*L*‐3′ (Kurath, 2012; Wolf, 1988). The unique *Nv*‐gene characterizes VHSV and the other members of its *Novirhabdovirus* genus, including *Salmonid novirhabdovirus* (=Infectious Hematopoietic Necrosis Virus [IHNV]), *Hirame novirhabovirus* (HIRRV), and *Snakehead novirhabdovirus* (SHRV); all infect fishes (Kurath, 2012). Studies of Rhabdoviridae evolution, including novirhabdoviruses (Getchell et al., 2017), have shown that they do not recombine and are inherited as a single unit (Walker et al., 2018); this facilitates resolution of their evolutionary patterns over time.

### 1.1 VHSV evolution, outbreaks, and hosts

Viral Hemorrhagic Septicemia Virus infects >140 species of fishes in marine, estuarine, and freshwater environments across the Northern Hemisphere, rendering it one of the world's most serious fish diseases (Escobar, Escobar‐Dodero, & Phelps, 2018). The evolutionary origin of VHSV has implicated a North Atlantic marine ancestor (Pierce & Stepien, 2012), whose lineage diversified into four genogroups (designated I–IV), with I–III found in the Northeastern Atlantic region of Europe (Hedrick et al., 2003; Meyers & Winton, 1995; Pierce & Stepien, 2012). VHSV genogroup I has a wide and diverse geographic range across Western Europe and has diversified into several subgenogroups (Pierce & Stepien, 2012). Genogroup I infects the most fish host species across a variety of estuarine and freshwaters (Kurath, 2012; Pierce & Stepien, 2012), including many aquacultured species (Abbadi et al., 2016; Ghorani et al., 2016). VHSV genogroup II diverged in Baltic Sea estuarine waters and is the sister group of genogroups I and III, with genogroup III mostly occurring in marine and estuarine waters of the North Sea (Pierce & Stepien, 2012; Stepien et al., 2020).

Genogroup IV comprises the sister group to the VHSV I–III (European) clade, with IV containing three allopatric subgenogroups (a–c) in North America (Pierce & Stepien, 2012; Stepien et al., 2020). In the 1980s, subgenogroup IVa emerged in coastal Northeastern Pacific waters, infecting salmonids and many marine fishes (Brunson, 1989; Hopper, 1989; Meyers et al., 1992). In 1996, IVa appeared in the Asian Northwestern Pacific, where it was apparently introduced from aquaculture trade (Takano, Nishizawa, Arimoto, & Muroga, 2000).

The Great Lakes' endemic subgenogroup—IVb—was back‐traced to its apparent origin in a 2003 muskellunge (*Esox masquinongy*) from Lake St. Clair (Ammayappan & Vakharia, 2009). The first outbreaks of IVb occurred during the spring months of 2005 and 2006, resulting in massive and widespread fish kills across the Great Lakes (Groocock et al., 2007; Lumsden et al., 2007; Thompson et al., 2011). In 2000, subgenogroup IVc was discovered in marine/estuarine North Atlantic waters (Gagné et al., 2007). IVc is the sister group of IVb, which together comprise a clade that is the sister group of IVa (Pierce & Stepien, 2012; Stepien et al., 2015, 2020).

From 2005 to 2008, widespread VHSV‐IVb outbreaks killed >32 fish species, whose external and internal hemorrhaging (Groocock et al., 2007; Lumsden et al., 2007; Whelan, 2007) caused the virus to be nicknamed “fish Ebola” (Hamblin, 2015). The virus then went “underground,” becoming less prevalent (Cornwell et al., 2015; Stepien et al., 2015, 2020). A smaller 2009 outbreak occurred in Lake St. Clair (Faisal et al., 2012), followed by scattered 2010 detections in Lakes Michigan, Huron, and Ontario, Budd Lake, and the St. Lawrence River (Cornwell et al., 2015; Faisal et al., 2012), and in two Lake Erie fishes in 2012 (Stepien et al., 2015). Single‐infected gizzard shad (*Dorosoma cepedianum*) individuals were reported from Lake Ontario in 2013 and the St. Lawrence River in 2014 (Getchell et al., 2017). In spring 2017, relatively minor and geographically restricted allopatric outbreaks occurred in Lake St. Clair (M. Faisal and G. Whelan, personal commination, 2017), Cayuga Lake (New York Finger Lakes), and Lake Ontario (Getchell et al., 2019; R. Getchell, personal communication, 2017). There have been no reported subsequent outbreaks.

VHSV‐IVb most often appears during spring water temperatures of 9–12°C (Escobar, Kurath, Escobar‐Dodero, Craft, & Phelps, 2017; Smail, 1999), coinciding with spawning of many of its host fish species (Scott & Crossman, 1973; Trautman, 1981), which may aid transmission (Stepien et al., 2015). Susceptibility varies among its fish host species. Freshwater drum (*Aplodinotus grunniens*) was severely affected in Lake Ontario's 2005 outbreak (Lumsden et al., 2007), and along with yellow perch (*Perca flavescens*) and largemouth bass (*Micropterus salmoides*) experienced large die‐offs in Lake Erie during the May–June 2006 outbreak (Kane‐Sutton, Kinter, Dennis, & Koonce, 2010; C. A. Stepien, personal observation, 2006). Round goby (*Neogobius melanostomus*), which was introduced to the Great Lakes in the 1990s (Jude, Reider, & Smith, 1992), also has undergone high VHSV‐IVb mortalities (Groocock et al., 2007; Lumsden et al., 2007). In 2010, large proportions of round goby tested positive, suggesting its persistent susceptibility and that it comprises a possible vector (Cornwell et al., 2014, 2015; Cornwell, Eckerlin, et al., 2012). The 2017 Cayuga Lake NY outbreak also occurred in round goby (Getchell et al., 2019).

It has been postulated that some invertebrates may harbor VHSV‐IVb, serving as possible reservoirs, including Hyalellidae amphipods (Throckmorton et al., 2017), Pontoporeiidae amphipods (e.g., *Diporeia* spp.; Faisal & Winters, 2011), and an individual leech (*Myzobdella lugubris*) (Faisal & Schulz, 2009). It has been suggested that zebra and quagga mussels (*Dreissena polymorpha* and *D. rostriformis*) might harbor the virus (M. Faisal, personal communication, 2015 and 2017). That possibility was investigated here, alongside broadscale fish population surveys. An invertebrate reservoir might pose increased threat to naïve areas via transfer or transport.

### 1.2 Aim and objectives

Our investigation aimed to evaluate whether VHSV‐IVb diversified during its two decades in the Great Lakes, and to elucidate possible spatial and temporal evolutionary patterns. The combined phylogenetic and population genetic approach analyzed partial sequences of glycoprotein (*G*‐gene) and 44 whole‐genome sequences (from a subset) from field‐collected samples of the virus' hosts, which had naturally occurring infections, using new samples, historic isolates, and sequences available in NCBI GenBank (https://www.ncbi.nlm.nih.gov/GenBank). We then related the results with patterns in other viruses, to augment understanding of virus and host coevolution.

## 2 MATERIALS AND METHODS

### 2.1 Sampling surveys

**FIGURE 2:**
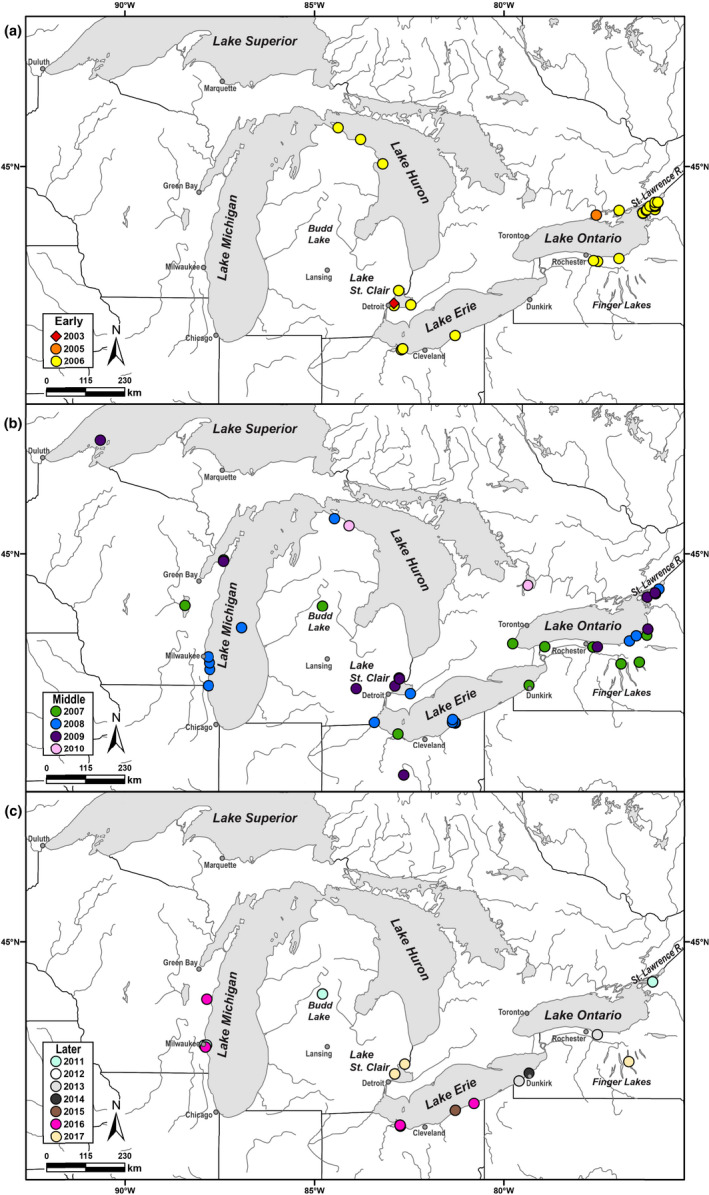
Maps showing locations (circles, colored by year) of VHSV‐IVb host samples and their isolates analyzed here, per time period (a) Early (2003–2006), (b) Middle (2007–2010), and (c) Later (2011–2017; none reported after 2017)

We sampled 55 fish species and 2,561 individuals in 2015 and 2016 (an interlude period) from 32 areas across the Great Lakes region, including locations of historic outbreaks and past positive records (Figure 2, Table 1). Water temperatures ranged from 2°C to 16.5°C, and multiple collection methods were employed (e.g., trawls, seines, gill nets, electrofishing) with federal, provincial, and state agency collaborators, following permits and regulations, and the University of Toledo Institutional Animal Care and Use Committee (IACUC) protocol #106419. In response to some 2017 outbreaks, we sampled 88 fishes in May–July from Lakes Erie, Michigan, and Ontario, and Cayuga Lake, as above, in water temperatures of 12–22°C. No outbreaks were reported or occurred during any of our samplings. Collection data included: date, method, depth, water temperature, GPS coordinates, species, total fish length (TL), sex (if determinable), and hemorrhages and/or other visual signs of disease.

**TABLE 1:** Field‐collected individuals surveyed and numbers of individuals that tested VHSV positive, their isolate numbers (note some individuals are designated with >1 system (per Niner, 2019, and Stepien et al., 2015, 2020), homology indicated by =), species' common and scientific names, total length (TL, mm), VHSV concentrations (molecules per actin molecules per our laboratory test in Pierce, Willey, Crawford, et al., 2013; Pierce, Willey, Palsule, et al., 2013; also see Figure 3), haplotypes (*G‐*gene and whole‐genome), and GenBank accession numbers from our field‐collected individuals in 2012, 2015, and 2016 (*N* = >1 individuals)

Sampling date	Sampling location (latitude, longitude)	*N* species surveyed (*N* VHSV positives)	*N* individuals surveyed (*N* VHSV positives) (isolate #)	Common name (scientific name)	TL (mm)	Concentration (VHSV/10^6^ actin)	*G*‐gene haplotype (GenBank)	Whole‐genome haplotype (GenBank)
4/12/2012	Lake Erie, Sandusky Bay, Sandusky, OH (41.453, −82.726)	1	1 (LMB‐12^a^)	Largemouth bass (*Micropterus salmoides*)	349	9.572E+02	k (MK777868)	N/A
5/10/2012	*“ “*	1	1 (FRD‐12^a^, =E06FD)	Freshwater drum (*Aplodinotus grunniens*)	625	1.117E+03	l (MK783004)	l (MK783004)
5/21/2015	Lake Erie, Fairport Harbor, OH (41.765259, −81.281594)	10 (2)	75 (2) (WPE‐15^a^, =WP‐H06)	White perch (*Morone americana*)	207	1.781E+03	u (MK777879)	N/A
5/26/2015	*“ “*	“ “	(ROG‐15^a^, =RGH‐31, =E15RG)	Round goby (*Neogobius melanostomus*)	179	5.244E+06	v (MK777880)	v (MK777880)
4/13/2016	Lake Erie, Sandusky Bay, Sandusky, OH (41.471594, −82.733653)	12 (4)	54 (13) (EMS‐16^a^, =B01)	Emerald shiner (*N* = 6, grouped) (*Notropis atherinoides*)	>75	8.448E+01	x (MK777881)	N/A
	*“ “*	“ “	(GIZ‐16‐9^a^ =B09^a^, =E16GSa)	Gizzard shad (*Dorosoma cepedianum*)	132	1.334E+05	w (MK783011)	w5 (MK783011)
	*“ “*	“ “	(GIZ‐16‐1^a^, =B11)	“ “	109	2.598E+02	w (MK777881)	N/A
	*“ “*	“ “	(GIZ‐16‐2^a^, =B14)	“ “	124	4.415E+04	“ “	N/A
	*“ “*	“ “	(GIZ‐16‐3^a^. =B10, = E16GSb)	“ “	130	7.754E+05	w (MK782997)	w4 (MK782997)
	*“ “*	“ “	(GIZ‐16‐4^a^, =B16)	“ “	119	2.980E+02	w (MK777881)	N/A
	*“ “*	“ “	(GIZ‐16‐6^a^, =B13, =E16GSc)	“ “	123	1.853E+03	w (MK782996)	w1 (MK782996)
	*“ “*	“ “	(GIZ‐16‐5^a^, =B19)	“ “	114	1.101E+01	w (MK777881)	N/A
	*“ “*	“ “	(GIZ‐16‐7^a^, =B17, =E16GSd)	“ “	109	1.875E+06	w (MK782994)	w2 (MK782994)
	*“ “*	“ “	(GIZ‐16‐8^a^, =B18, =E16GSe)	“ “	436	7.764E+01	w (MK782995)	w3 (MK782995)
	*“ “*	“ “	(PUM‐16^a^ =B20)	Pumpkinseed (*Lepomis gibbosus*)	134	3.388E+00	w (MK777881)	N/A
	*“ “*	“ “	(LMB‐16‐1^a^, =B21)	Largemouth bass (*Micropterus salmoides*)	156	5.050E+01	“ “	N/A
	“ “	“ “	(LMB‐16‐2^a^, =B22)	“ “	149	1.399E+01	“ “	N/A
5/18/2016	Lake Erie, Ashtabula, OH (42.995682, −87.81905)	16 (1)	72 (1) (LMB‐16‐3^a^, G61*)	“ “	340	1.199E+01	“ “	N/A
5/25–26/2016	Lake Michigan, South Shore Park Milwaukee, WI (42.995682, −87.881905)	6 (2)	100 (5) (ROG‐16‐1^a^, =L56, =M16RGa)	Round goby (*N* = 3, grouped)	<80	7.018E+02	x (MK783001)	x1 (MK783001)
		“ “	(ROG‐16‐2^a^ =L59, =M16RGb)	Round goby (*N* = 4, grouped)	<80	5.706E+03	x (MK783000)	x2 (MK783000)
5/26/2016	Lake Michigan, Grant Park Milwaukee, WI (42.920696, −87.846161)	“ “	(ROG‐16‐3^a^, =L72)	Round goby	73	5.465E+01	x (MK777885)	N/A
			(ROG‐16‐4^a^, =L73)	“ “	66	2.722E+01	“ “	N/A
“ “	Lake Michigan, South Shore Park Milwaukee, WI (42.995682, −87.881905)	“ “	(ALE‐16^a^, =L75)	Alewife (*Alosa pseudoharengus*)	98	5.104E+01	“ “	N/A

^a^ Individuals assayed for VHSV concentration here and in Figure 3. “ “ = same as above.

Fishes were euthanized by an overdose of 25 mg/ml tricaine methanesulfonate (MS‐222; Argent Chemical Laboratories) and sacrificed according to the University of Toledo IACUC #106419. Individual surgical sites (anus–operculum) each were cleaned with alcohol wipes, and a new sterile razor blade was used; the spleen was removed using sterile forceps and placed in a sterile labeled tube containing 1.5 ml RNAlater (Qiagen). Each liver was wrapped individually in sterile aluminum foil, and archived at −80°C. Spleen and liver tissues later were pooled in single tubes for up to five individuals (<50 mm TL) of the same species, collection, and location. Between specimens, forceps and aluminum dissection trays were sterilized, razor blades discarded in sharps containers, and gloves changed. Specimen disposal followed the respective sampling agency protocol and/or UT IACUC#106419.

In 2015, to examine potential harboring of IVb, 50–100 dreissenid mussels were collected from all sites except for Budd Lake, where sufficient numbers occurred, and tested for VHSV. Each mussel was smashed on a sterile surface with the back end of sterile forceps. Half of each was placed in RNAlater (as above), and the other half was wrapped in sterile foil and stored at −80°C.

### 2.2 RNA extraction and reverse transcription

Tissues from up to 11 individuals per location were pooled in sterile 1.5 ml tubes for initial processing and ≤0.5 g ground with a sterilized mortar and pestle under liquid nitrogen. RNA was extracted with the Trizol^®^ (Molecular Research Center, Inc.) protocol, resuspended in 30 µl RNase‐free water, and quantified with a NanoDrop 2000 spectrophotometer (Thermo Fisher Scientific). Reverse transcription to complementary (c)DNA was performed by first incubating 1 μg RNA with 100 ng of random hexamer primer in 7 μl at 65°C for 10 min. Reactions were cooled to 4°C before adding 13 μl M‐MLV‐RT mixture (10× First Stand buffer [Ambion Life Technologies], 10 mM dNTPs, 0.05 mM random hexamers, 25 U/µl RNasin, and 200 U/µl M‐MLV) and incubated at 42°C for 1 hr following Stepien et al. (2015). cDNA was labeled and stored at −80°C.

### 2.3 qPCR tests for VHSV‐IVb and quantification

Presence or absence of VHSV‐IVb was determined using the Stepien laboratory's SYBR^®^ Green quantitative PCR assay protocol (Pierce, Willey, Crawford, et al., 2013) for the *N*‐gene. Cell culture standards spiked with known titers (0, 10, 1 × 10, 1 × 10^3^, and 1 × 10^4^ pfu RNA/ml) provided reference VHSV‐IVb readings. To circumvent false negatives, simultaneous control β‐actin housekeeping gene assays were run alongside VHSV reactions. Runs contained a positive β‐actin/negative virus control (0 pfu/ml), low concentration of the virus (10 pfu), high virus control (1 × 10^4^ pfu), and nuclease‐free water as a negative control. Pooled samples were tested in triplicate for β‐actin and VHSV. Individual samples from pools reading VHSV positive (*C*_t,_ <35) were extracted and assayed, to pinpoint individual isolates.

Following identification of positive samples, their cDNA was quantified for number of VHSV versus β‐actin molecules using the two‐color fluorometric real‐time PCR (2CF‐qPCR) assay developed by the Stepien laboratory (Pierce, Willey, Palsule, et al., 2013). This employs two internal standards (IS), respectively, for the VHSV‐IVb *N*‐gene and the β‐actin gene (Pierce, Willey, Palsule, et al., 2013), whose output diagnoses among three alternatives: (a) reaction failure (no amplification), (b) VHSV negative (amplification of VHSV IS alone), or (c) VHSV positive (amplification of both the sample and IS). Variable ratios of VHSV and β‐actin IS are used to quantify the products (Pierce, Willey, Crawford, et al., 2013), with reactions run in triplicate, reporting means and standard errors. Viral levels were compared to prior haplotype “a” challenge experiment results (Pierce, Willey, Palsule, et al., 2013).

### 2.4 Preparation of historic isolates in cell culture

Samples from historic VHSV outbreaks (2006–2011) were received from G. Kurath (USGS, Seattle, WA) as frozen media from BF2 cell culture or as RNA, to which 30 and 150 µl of serum‐free MEM (minimum essential media; Thermo Fisher Scientific) were added per well of a plate confluent with BF2 cells. Cells were incubated with media at 20°C for 1 hr, after which media were replaced with 10% serum MEM, and incubated at 20°C for <1 week. At ≥80% cytopathogenicity (CPE), media were collected in a 1.5 ml tube, 250 µl versene added for 10 min, centrifuged for 4 min at 1,700 *g* at 4°C, and the supernatant discarded. We added 250 µl Trizol^®^ to the cell solids, and isolated RNA and cDNA as above. All samples were passaged twice, to minimize possible NT mutations during cell culture. We were successful at sequencing the whole genome from 40 isolates.

### 2.5 Sequencing VHSV isolates

cDNA was synthesized from total RNA extracted from tissue samples using SuperScript IV (Invitrogen), following manufacturer's instructions. Genomic cDNA was amplified in four segments using primers from Schönherz, Lorenzen, Guldbrandtsen, Buitenhuis, and Einer‐Jensen (2016), substituting VHSV_Frag1I_nt18_+s with a more specific primer designed by us (5′GAGAGCTGCAGCACTTCACCG C3′), and 1 μl cDNA in 25 µl reactions with One Taq DNA polymerase (New England Biolabs). Positive controls of “a” were resequenced to confirm accuracy, and nuclease‐free ddH_2_O served as a negative control. Any and all NT differences from “a” in the historic and new samples were confirmed with corresponding trace files. Amplicons were examined under UV light on 1% agarose gels stained with ethidium bromide. Target PCR products were gel excised and purified using QIAquick Gel Extraction kits (Qiagen).

Additional PCRs amplified the front 700 nucleotides (NTs) and end 400 NTs of the genome, with 45 s extension time. The front segment utilized VHSV_Frag1I_nt18_+s (5′GAGTTATGTTACARGGGACAGG3′) (Schönherz et al., 2016) and antisense (5′TGACCGAGATGGCAGATC3′), and end primers were designed based on the VHSV‐IVb genome (GenBank: GQ385941) (End sense: 5′CCCAGATGCTATCACCGAGAA3′, End antisense: 5′ACAAAGAATCCGAGGCAGGAG3′). Cleaned products were Sanger sequenced at Cornell DNA Services, and the sequences were aligned and analyzed by us using MEGA X (Kumar, Stecher, Li, Knyaz, & Tamura, 2018).

Genomic sequencing was performed at Ohio State University's Molecular and Cellular Imaging Center (Wooster, OH). Sequences were uploaded by us to the Galaxy web platform, and analyzed with usegalaxy.org programs (Afgan et al., 2018). Segments were aligned to the reference VHSV‐IVb genome (C03MU, GenBank: GQ385941) using MAP WITH BWA‐MEM (Li, 2013). For each of the isolates, consensus sequences were generated followed by manual checking of each single‐nucleotide polymorphism (SNP) and coverage read using Integrative Genomics Viewer (IGV: Robinson et al., 2011; Thorvaldsdóttir, Robinson, & Mesirov, 2013). Consensus sequences, front, and end segments were concatenated, aligned, and trimmed in MEGA X.

Haplotypes are defined here as “unique gene sequences that differ by one or more NT substitutions” from haplotype “a,” which was the original 2003 isolate MI03GL sequenced from a Lake St. Clair muskellunge (GQ385941) (see Table 2). Haplotypes from the whole genomes were designated with their *G*‐gene designations followed by a number.

**TABLE 2:** VHSV‐IVb samples used in our analyses. Isolate name (and alternate name from Niner, 2019, and Stepien et al., 2020, used for whole‐genome sequences), year, location information, geographic coordinates, host species, haplotype, GenBank accession number, and gene dataset (*G*‐gene or All = whole genome) are provided for the (1) Early, (2) Middle, and (3) Later time periods

Isolate name	Year	Body of water	Region	Nearest city	Lat, long	Host species	Haplotype	Accession no.	Gene data set
**1. Early**									
TAVgr05‐01 (C03MU)	2003	L. St. Clair	Central	Detroit, MI	42.391, −82.911	Muskellunge (*Esox masquinongy*)	a	GQ385941, KY359354	*G*/All
U 13653‐1	2005	L. Ontario	Lower	Brighton, ON	43.968, −77.629	Freshwater drum (*Aplodinotus grunniens*)	b	HQ453209	*G*
U 13653‐2	“ “	“ “	“ “	“ “	“ “	“ “	“ “	“ “	“ “
5464 Bluegill	2006	L. St. Clair	Upper	Jeannette's Creek, ON	42.358, −82.459	Bluegill (*Lepomis macrochirus*)	a	GQ385941	“ “
5464 Drum	“ “	“ “	“ “	“ “	“ “	Freshwater drum	“ “	“ “	“ “
5464 Smallmouth bass	“ “	“ “	“ “	“ “	“ “	Smallmouth bass (*Micropterus dolomieu*)	“ “	“ “	“ “
Goby 1‐5	“ “	L. Ontario	Lower	Cape Vincent, NY	44.126, −76.334	Round goby (*Neogobius melanostomus*)	b	AB672615	*G*
TAVgr06‐01	“ “	“ “	“ “	Rochester, NY	43.216, −77.633	“ “	“ “	HQ453209	*“ “*
TAVgr06‐02	“ “	St. Lawrence R.	“ “	Clayton, NY	44.250, −76.016	Muskellunge	“ “	“ “	“ “
TAVgr06‐03 (=O06RG)	“ “	“ “	“ “	Cape Vincent, NY	44.127, −76.333	Round goby	“ “	EF564588, KY359357	*G*/All
TAVgr06‐04	“ “	“ “	“ “	“ “	“ “	“ “	“ “	“ “	“ “
TAVgr06‐05	“ “	L. Erie	Central	Sandusky, OH	41.474, −82.703	Freshwater drum	a	GQ385941	“ “
TAVgr06‐06	“ “	“ “	“ “	“ “	“ “	“ “	“ “	“ “	“ “
TAVgr06‐07 (=E06FD)	“ “	“ “	“ “	Fairport Harbor, OH	41.756, −81.287	“ “	a1	MK783014	*G*/All
TAVgr06‐08 (=E06WA)	“ “	“ “	“ “	“ “	“ “	Walleye (*Sander vitreus*)	a2	MK782987	*G*/Al
TAVgr06‐09 (=E06WBc)	“ “	“ “	“ “	“ “	“ “	White bass (*Morone chrysops*)	a	MK782986	*G*/All
TAVgr06‐10 (=E06YPa)	“ “	“ “	“ “	“ “	“ “	Yellow perch (*Perca flavescens*)	a10	MK782985	*G*/All
TAVgr06‐11 (=E06SB)	“ “	“ “	“ “	“ “	“ “	Smallmouth bass	a3	MK782984	*G*/All
TAVgr06‐12 (=E06YPb)	“ “	“ “	“ “	Sandusky, OH	41.492, −82.667	Yellow perch	a13	MK782983	*G*/All
TAVgr06‐13 (=E06YPc)	“ “	“ “	“ “	“ “	“ “	“ “	a14	MK782982	*G*/All
TAVgr06‐14 (=E06WBb)	“ “	“ “	“ “	“ “	“ “	White bass	a4	MK783013	*G*/All
TAVgr06‐15	“ “	“ “	“ “	Fairport Harbor, OH	41.755, −81.286	Gizzard shad (*Dorosoma cepedianum*)	a	GQ385941	*G*
TAVgr06‐16 (=E06WBa)	“ “	“ “	“ “	Sandusky, OH	41.492, −82.667	White bass	d	MK777861	*G*/All
TAVgr06‐17	“ “	“ “	“ “	“ “	“ “	Walleye	a	GQ385941	*G*
TAVgr06‐18	“ “	“ “	“ “	“ “	“ “	Emerald shiner (*Notropis atherinoides*)	“ “	“ “	“ “
TAVgr06‐19	“ “	St. Lawrence R.	Lower	Wellesley Island, NY	44.327, −75.937	Brown bullhead (*Ameiurus nebulosus*)	b	HQ453209	“ “
TAVgr06‐20	“ “	“ “	“ “	“ “	44.323, −75.935	White perch (*Morone americana*)	“ “	“ “	“ “
TAVgr06‐21	“ “	“ “	“ “	Clayton, NY	44.248, −76.098	Northern pike (*Esox lucius*)	“ “	“ “	“ “
TAVgr06‐22	“ “	“ “	“ “	Grindstone, NY	44.254, −76.150	Brown bullhead	“ “	“ “	“ “
TAVgr06‐23	“ “	“ “	“ “	Cape Vincent, NY	44.187, −76.225	Bluegill	“ “	“ “	“ “
TAVgr06‐24	“ “	“ “	“ “	“ “	44.172, −76.247	Yellow perch	“ “	“ “	“ “
TAVgr06‐25	“ “	“ “	“ “	Alexandria, NY	44.323, −75.935	“ “	“ “	“ “	“ “
TAVgr06‐26	“ “	“ “	“ “	Cape Vincent, NY	44.187, −76.225	Smallmouth bass	“ “	“ “	“ “
TAVgr06‐27	“ “	“ “	“ “	Clayton, NY	44.250, −76.016	Burbot (*Lota lota*)	“ “	“ “	“ “
TAVgr06‐28	“ “	L. Ontario	“ “	Irondequoit, NY	43.200, −77.527	Round goby	a	MK777874	*G*
TAVgr06‐29	“ “	“ “	“ “	Sodus Bay, NY	43.249, −76.962	Smallmouth bass	“ “	GQ385941	*“ “*
TAVgr06‐30	“ “	“ “	“ “	Cape Vincent, NY	44.116, −76.333	“ “	b	HQ453209	*G*
TAVgr06‐31	“ “	St. Lawrence R.	“ “	Clayton, NY	44.251, −76.134	Rock bass (*Ambloplites rupestris*)	“ “	“ “	*“ “*
TAVgr06‐32	“ “	“ “	“ “	Wellesley Island, NY	44.323, −76.014	Black crappie (*Pomoxis nigromaculatus*)	“ “	“ “	“ “
TAVgr06‐33	“ “	“ “	“ “	“ “	“ “	“ “	“ “	“ “	“ “
TAVgr06‐34	“ “	“ “	“ “	Clayton, NY	44.248, −76.014	Smallmouth bass	“ “	“ “	“ “
TAVgr06‐35	“ “	“ “	“ “	“ “	44.254, −76.014	*Ictalurus punctatus*	“ “	“ “	“ “
TAVgr06‐36	“ “	“ “	“ “	“ “	44.187, −76.014	“ “	“ “	“ “	“ “
TAVgr06‐37	“ “	“ “	“ “	Greater Napanee, ON	44.172, −76.964	Largemouth bass (*Micropterus salmoides*)	“ “	“ “	“ “
TAVgr06‐38	“ “	“ “	“ “	Wellesley Island, NY	44.323, −76.014	Smallmouth bass	“ “	“ “	“ “
TAVgr06‐39	“ “	“ “	“ “	Clayton, NY	44.187, −76.014	“ “	“ “	“ “	“ “
TAVgr06‐40	“ “	“ “	“ “	Orleans, NY	44.268, −76.014	“ “	“ “	“ “	“ “
TAVgr06‐43	“ “	L. Huron	Upper	Alpena, MI	45.050, −83.200	Walleye	a	GQ385941	“ “
TAVgr06‐44	“ “	“ “	“ “	Rogers City, MI	45.502, −83.783	Chinook salmon (*Oncorhynchus tshawytscha*)	“ “	“ “	“ “
TAVgr06‐45	“ “	“ “	“ “	Alpena, MI	45.050, −83.200	Lake whitefish (*Coregonus clupeaformis*)	“ “	“ “	“ “
TAVgr06‐46	“ “	“ “	“ “	Bois Blanc, MI	45.718, −84.374	“ “	“ “	“ “	“ “
TAVgr06‐47 (=C06NP)	“ “	L. St. Clair	Central	Harrison Township, MI	42.635, −82.778	Northern pike	“ “	MK782990	*G*/All
TAVgr06‐48 (=C06GS)	“ “	“ “	“ “	Grosse Point, MI	42.343, −82.902	Gizzard shad	a6	MK777875	*G*/All
TAVgr06‐49 (=C06RB)	“ “	“ “	“ “	“ “	“ “	Rock bass	a	GQ385941, MK782990	*G*/All
TAVgr06‐50 (=C06SR)	“ “	“ “	“ “	“ “	“ “	Shorthead redhorse (*Moxostoma macrolepidotum*)	“ “	“ “	*G*/All
TAVgr06‐51 (=C06YP)	“ “	“ “	“ “	“ “	“ “	Yellow perch	“ “	“ “	*G*/All
TAVgr06‐52 (=C06FD)	“ “	“ “	“ “	“ “	“ “	Freshwater drum	“ “	“ “	*G*/All
TAVgr06‐53	“ “	“ “	“ “	“ “	“ “	Trout‐perch *Percopsis omiscomaycus*	m	MEAP VHSV database	*G*
**2. Middle**									
OMNR #5577	2007	L. Ontario	Lower	Hamilton Beach, ON	43.295, −79.772	Freshwater drum	a	HQ623440	*“ “*
OMNR #5583	“ “	“ “	“ “	“ “	“ “	*“ “*	“ “	“ “	“ “
OMNR #5579	“ “	L. St. Clair	Central	Jeannette's Creek, ON	42.328, −82.472	Largemouth bass	“ “	“ “	“ “
TAVgr07‐01 (=B0BG)	“ “	Budd L.	Upper	Harrison, MI	44.016, −84.788	Bluegill	e	MK783006, MK772862	*G*/All
TAVgr07‐02	“ “	“ “	“ “	“ “	“ “	Black crappie	a	HQ623440	*G*
TAVgr07‐03	“ “	“ “	“ “	“ “	“ “	Largemouth bass	“ “	“ “	“ “
TAVgr07‐04 (=B07PS)	“ “	“ “	“ “	“ “	“ “	Pumpkinseed (*Lepomis gibbosus*)	f	MK783008, MK777863	*G*/All
TAVgr07‐05	“ “	L. Michigan	Upper	Oshkosh, WI	44.028, −88.421	Freshwater drum	a	HQ623440	*G*
TAVgr07‐06	“ “	L. Ontario	Lower	Irondequoit, NY	43.233, −77.650	Gizzard shad	“ “	“ “	“ “
TAVgr07‐07	“ “	“ “	“ “	“ “	“ “	*“ “*	“ “	“ “	“ “
TAVgr07‐08	“ “	L. Erie	Central	Dunkirk, NY	42.490, −79.338	*“ “*	b	HQ453209	“ “
TAVgr07‐09	“ “	“ “	“ “	“ “	“ “	*“ “*	a	HQ623440	“ “
TAVgr07‐10	“ “	L. Ontario	Lower	Ransomville, NY	43.240, −79.920	Black crappie	“ “	“ “	*G*
TAVgr07‐11	“ “	“ “	“ “	“ “	“ “	*“ “*	“ “	“ “	“ “
TAVgr07‐12 (=E07CC)	“ “	L. Erie	Central	Dunkirk, NY	42.491, −79.338	Common carp (*Cyprinus carpio*)	c	MK783005, MK777860	*G*/All
TAVgr07‐13	“ “	L. Ontario	Lower	Skaneateles, NY	42.939, −76.425	Lake trout (*Salvelinus namaycush*)	“ “	MK777860	*G*
TAVgr07‐14	“ “	“ “	“ “	Waterloo, NY	42.910, −76.910	Pumpkinseed	a	HQ623440	“ “
TAVgr07‐15	“ “	“ “	“ “	“ “	“ “	*“ “*	“ “	“ “	“ “
TAVgr07‐16	“ “	“ “	“ “	“ “	“ “	*“ “*	“ “	“ “	“ “
TAVgr07‐17	“ “	“ “	“ “	Mexico, NY	43.459, −76.228	Rainbow trout (*Oncorhynchus mykiss*)	c	HQ623435	“ “
TAVgr07‐18	“ “	“ “	“ “	Skaneateles, NY	42.939, −76.425	Smallmouth bass	b	HQ453209	“ “
TAVgr07‐19	“ “	“ “	“ “	“ “	“ “	Rock bass	“ “	“ “	“ “
TAVgr07‐20	“ “	L. Erie	Central	Catawba Island, OH	41.542, −82.789	Bluegill	g	MK777864	*“ “*
TAVgr07‐21 (=E07YPa)	“ “	“ “	“ “	Fairport Harbor, OH	41.801, −81.356	Yellow perch	a7	MK782989	*G*/All
TAVgr07‐22 (=E07YPb)	“ “	“ “	“ “	“ “	41.755, −81.277	*“ “*	a5	MK782988	*G*/All
TAVgr07‐24 (=M07SB)	“ “	L. Michigan	Upper	Sturgeon Bay, WI	44.885, −87.389	Smallmouth bass	h	MK783009	*G*/All
TAVgr08‐02	2008	“ “	“ “	Racine, WI	42.799, −87.761	Yellow perch	b	MK777859	*G*
TAVgr08‐03 (=M08RB)	“ “	“ “	Upper	Kenosha, WI	42.485, −87.800	Rock bass	i	MK783010	*G*/All
TAVgr08‐04	“ “	St. Lawrence R.	Lower	Clayton, NY	44.250, −76.016	Round goby	b	HQ453209	*G*
TAVgr08‐05	“ “	L. Ontario	“ “	Oswego, NY	43.450, −76.510	“ “	“ “	“ “	“ “
TAVgr08‐06	“ “	“ “	“ “	Sterling, NY	43.350, −76.690	“ “	“ “	“ “	“ “
TAVgr08‐07	“ “	St. Lawrence R.	“ “	St. Lawrence River	43.340, −75.910	“ “	“ “	“ “	“ “
TAVgr08‐08	“ “	L. Huron	Upper	Cheboygan, MI	45.652, −84.468	Sea lamprey (*Petromyzon marinus*)	“ “	“ “	“ “
TAVgr08‐09 (=E08ES)	“ “	L. Erie	Central	Fairport Harbor, OH	41.769, −81.294	Emerald shiner	a8	MK783012	*G*/All
TAVgr08‐10 (=E08FDa)	“ “	“ “	“ “	“ “	41.769, −81.354	Freshwater drum	a15	MK782993	*G*/All
TAVgr08‐11 (=E08FDb)	“ “	“ “	“ “	“ “	“ “	“ “	a11	MK782992	*G*/All
TAVgr09‐01 (M08AMa)	“ “	Lake Michigan	Upper	Benona Township, MI	43.600, −86.916	Amphipod	a	MK782990	*G*/All
TAVgr09‐02 (M08AMb)	“ “	“ “	“ “	“ “	“ “	“ “	“ “	“ “	*G/*All
TAVgr09‐03 (C08LEa)	“ “	Lake St. Clair	Central	Chesterfield Township, MI	42.631, −82.765	Leech	]“ “	“ “	*G*/All
TAVgr09‐04 (C08LEb)	“ “	“ “	“ “	“ “	“ “	“ “	“ “	“ “	*G*/All
TAVgr09‐05	“ “	L. Erie	“ “	Lake Erie	N/A	“ “	“ “	“ “	*G*
TAVgr09‐13	“ “	L. Michigan	Upper	Milwaukee, WI	42.926, −87.770	Round goby	“ “	HQ643440	*“ “*
TAVgr09‐17 (=M08YP)	“ “	“ “	“ “	“ “	43.040, −87.802	Yellow perch	j	MK783007	*G*/All
TAVgr09‐09	2009	Baseline Lake	“ “	Pinckney, MI	42.427, −83.899	Brown bullhead	a	HQ623441	*G*
TAVgr09‐10 (=C09MU)	“ “	L. St. Clair	Central	Harrison Township, MI	42.616, −82.757	Muskellunge	“ “	MK782990	*G*/All
TAVgr09‐11	“ “	“ “	“ “	St. Clair Shores, MI	42.475, −82.879	Smallmouth bass	“ “	HQ643441	*G*
TAVgr10‐01	“ “	L. Superior	Upper	Apostle Islands, WI	47.085, −90.641	Cisco (*Coregonus artedi*)	“ “	“ “	“ “
TAVgr10‐05	“ “	Lake Ontario	Lower	Irondequoit, NY	43.236, −77.534	Round goby	“ “	“ “	“ “
TAVgr10‐06	“ “	“ “	“ “	Pulaski, NY	43.577, −76.203	“ “	b	HQ453209	“ “
TAVgr10‐07	“ “	“ “	“ “	“ “	“ “	“ “	o	MEAP VHSV database	“ “
TAVgr10‐08	“ “	“ “	“ “	“ “	“ “	“ “	p	“ “	“ “
TAVgr10‐09	“ “	St. Lawrence R.	“ “	Clayton, NY	44.266, −76.012	“ “	b	HQ453209	“ “
TAVgr10‐10	“ “	“ “	“ “	Cape Vincent, NY	44.186, −76.224	“ “	p	MEAP VHSV database	“ “
TAVgr10‐11	“ “	L. Ontario	“ “	Pulaski, NY	43.577, −76.203	“ “	o	“ “	“ “
TAVgr10‐12	“ “	“ “	“ “	“ “	“ “	“ “	q	“ “	“ “
TAVgr9‐12	“ “	Lake Michigan	Upper	Sturgeon Bay, WI	44.860, −87.393	Smallmouth bass	s	HQ623440	“ “
GL2010‐098	2010	L. Huron	“ “	Barrie, ON	44.407, −79.368	Round goby	“ “	“ “	“ “
TAVgr10‐02	“ “	“ “	“ “	Huron Beach, MI	45.519, −84.087	Lake trout	n	MEAP VHSV database	“ “
**3. Later**									
TAVgr11‐01	2011	L. Michigan	Upper	Milwaukee, WI	43.030, −87.915	Gizzard shad	r	“ “	*G*
TAVgr11‐02	“ “	“ “	“ “	“ “	“ “	“ “	“ “	“ “	“ “
TAVgr11‐18	“ “	“ “	“ “	“ “	43.036, −87.853	Yellow perch	a	HQ453209	“ “
TAVgr11‐19 (=M11YP)	“ “	“ “	“ “	“ “	“ “	“ “	a9	MK782991	*G*/All
TAVgr11‐03	“ “	Budd L.	“ “	Harrison, MI	44.015, −84.788	Smallmouth bass	a	HQ453209	*G*
TAVgr11‐05	“ “	“ “	“ “	“ “	“ “	Largemouth bass	“ “	“ “	“ “
TAVgr11‐08	“ “	“ “	“ “	“ “	“ “	“ “	“ “	“ “	“ “
TAVgr11‐09	“ “	“ “	“ “	“ “	“ “	“ “	“ “	“ “	“ “
TAVgr11‐11	“ “	“ “	“ “	“ “	“ “	“ “	“ “	“ “	“ “
TAVgr11‐13	“ “	“ “	“ “	“ “	“ “	“ “	“ “	“ “	“ “
TAVgr11‐15	“ “	“ “	“ “	“ “	“ “	“ “	“ “	“ “	“ “
TAVgr11‐17	“ “	“ “	“ “	“ “	“ “	“ “	“ “	“ “	“ “
vcG017	“ “	St. Lawrence R.	Lower	Clayton, NY	44.243, −76.079	Round goby		Cornwell et al. (2014)	“ “
“ “	“ “	“ “	“ “	“ “	“ “	“ “	“ “	“ “	“ “
vcG018	“ “	“ “	“ “	“ “	“ “	“ “	z	“ “	“ “
“ “	“ “	“ “	“ “	“ “	“ “	“ “	“ “	“ “	“ “
“ “	“ “	“ “	“ “	“ “	“ “	“ “	“ “	“ “	“ “
“ “	“ “	“ “	“ “	“ “	“ “	“ “	“ “	“ “	“ “
vcG001	“ “	“ “	“ “	“ “	“ “	“ “	a	HQ453209	“ “
vcG002	“ “	“ “	“ “	“ “	“ “	“ “	b	EF564588.1	“ “
“ “	“ “	“ “	“ “	“ “	“ “	“ “	“ “	“ “	“ “
“ “	“ “	“ “	“ “	“ “	“ “	“ “	“ “	“ “	“ “
“ “	“ “	“ “	“ “	“ “	“ “	“ “	“ “	“ “	“ “
“ “	“ “	“ “	“ “	“ “	“ “	“ “	“ “	“ “	“ “
“ “	“ “	“ “	“ “	“ “	“ “	“ “	“ “	“ “	“ “
“ “	“ “	“ “	“ “	“ “	“ “	“ “	“ “	“ “	“ “
vcG032	“ “	“ “	“ “	“ “	“ “	“ “	aa	Cornwell et al. (2014)	“ “
“ “	“ “	“ “	“ “	“ “	“ “	“ “	“ “	“ “	“ “
vcG045	“ “	“ “	“ “	“ “	“ “	“ “	ab	“ “	“ “
vcG046	“ “	“ “	“ “	“ “	“ “	“ “	ac	“ “	“ “
vcG047	“ “	“ “	“ “	“ “	“ “	“ “	ad	“ “	“ “
vcG048	“ “	“ “	“ “	“ “	“ “	“ “	ae	“ “	“ “
vcG049	“ “	“ “	“ “	“ “	“ “	“ “	af	“ “	“ “
vcG050	“ “	“ “	“ “	“ “	“ “	“ “	ag	“ “	“ “
“ “	“ “	“ “	“ “	“ “	“ “	“ “	“ “	“ “	“ “
“ “	“ “	“ “	“ “	“ “	“ “	“ “	“ “	“ “	“ “
“ “	“ “	“ “	“ “	“ “	“ “	“ “	“ “	“ “	“ “
vcG051	“ “	“ “	“ “	“ “	“ “	“ “	ah	“ “	“ “
FRD‐12 (=E12FD)	2012	L. Erie	Central	Sandusky, OH	41.453, −82.726	Freshwater drum	l	MK783004	*G*/All
LMB‐12	“ “	“ “	“ “	“ “	“ “	Largemouth bass	k	MK777868	*G*
RPL2013‐002 (=O13GS)	2013	L. Ontario	Lower	Irondequoit Bay, NY	43.231, −77.533	Gizzard shad	bc	KY359355 (Getchell et al., 2017)	*G*/All
FPL2014‐001 (=E14GS)	2014	L. Erie	Central	Dunkirk, NY	42.495, −79.333	“ “	bd	KY359356 (Getchell et al., 2017)	*G*/All
RG‐H31 (=ROG‐15,=E15RG)	2015	“ “	“ “	Fairport Harbor, OH	41.765, −81.281	Round goby	v	MK783003	*G*/All
WP‐H06 (=WPE‐15)	“ “	“ “	“ “	“ “	“ “	White perch	u	MK777879	*G*
B01	2016	“ “	“ “	Sandusky, OH	41.471, −82.733	Emerald shiner (*N. atherinoides*)	x	MK777881	*“ “*
B09 (=GIZ16‐9, =E16GSa)	“ “	“ “	“ “	“ “	“ “	Gizzard shad	w5	MK783011	*G*/All
B10 (=GIZ16‐3, =E16GSb)	“ “	“ “	“ “	“ “	“ “	“ “	w4	MK782997	*G*/All
B11 (=GIZ16‐1)	“ “	“ “	“ “	“ “	“ “	“ “	w	MK777881	*G*
B13 (=GIZ16‐6, =E16GSc)	“ “	“ “	“ “	“ “	“ “	“ “	w1	MK782996	*G*/All
B14 (=GIZ16‐2)	“ “	“ “	“ “	“ “	“ “	“ “	w	MK77881	*G*
B16 (=GIZ16‐4)	“ “	“ “	“ “	“ “	“ “	“ “	“ “	“ “	*“ “*
B17 (=GIZ‐16‐7, =E16GSd)	“ “	“ “	“ “	“ “	“ “	“ “	w2	MK782994	*G*/All
B18 (=GIZ‐16‐8, =E16GSe)	“ “	“ “	“ “	“ “	“ “	“ “	w3	MK782995	*G*/All
B19 (=GIZ‐16‐5)	“ “	“ “	“ “	“ “	“ “	“ “	w	MK777881	*G*
B20 (=PUM‐16)	“ “	“ “	“ “	“ “	“ “	Pumpkinseed	“ “	“ “	“ “
B21	“ “	“ “	“ “	“ “	“ “	Largemouth bass	“ “	“ “	“ “
B22	“ “	“ “	“ “	“ “	“ “	“ “	“ “	“ “	“ “
G61	“ “	“ “	“ “	Ashtabula, OH	41.898, −80.795	“ “	“ “	“ “	*“ “*
L56 (=M16RGa)	“ “	L. Michigan	Upper	Milwaukee, WI	42.996, −87.882	Round goby	x1	MK783001	*G*/All
L59 (=M16RGb)	“ “	“ “	“ “	“ “	“ “	“ “	x2	MK783000	*G*/All
L75 (=ALE‐16)	“ “	“ “	“ “	“ “	“ “	Alewife (*Alosa pseudoharengus*)	x	MK777885	*G*
L72 (=ROG‐16‐3)	“ “	“ “	“ “	“ “	43.920, −87.846	Round goby	“ “	“ “	*“ “*
L73 (=ROG‐16‐4)	“ “	“ “	“ “	“ “	“ “	“ “	“ “	“ “	“ “
1704122	2017	L. St. Clair	Central	St. Clair Shores, MI	42.473, −82.880	Gizzard shad	be	M. Faisal, Personal communication (2017)	*“ “*
1704123	“ “	“ “	“ “	“ “	“ “	“ “	“ “	“ “	“ “
1704124	“ “	“ “	“ “	“ “	“ “	“ “	“ “	“ “	“ “
1704125	“ “	“ “	“ “	“ “	“ “	Black crappie	“ “	“ “	“ “
1703302	“ “	“ “	“ “	Clay Township, MI	42.663, −82.617	Bluegill	“ “	“ “	“ “

“ “ = same as above.

### 2.6 Genetic data analyses

We compiled our 44 *G*‐gene sequence isolates with an additional 140 from NIH GenBank (https://www.ncbi.nlm.nih.gov/GenBank), sourcing the 2013 database (http://gis.nacse.org/vhsv/) (*N* = 114), Cornwell et al. (2014) (*N* = 10), Stepien et al. (2015) (*N* = 11), Getchell et al. (2017) (*N* = 4), and M. Faisal and G. Whelan (personal communication, 2017) (*N* = 1), totaling 184 VHSV‐IVb sequences for the 669 NT central *G*‐gene segment. The *G*‐gene was our primary focus for the population genetic analyses, due to sequence availability and more robust sample sizes. Results were compared with 40 VHSV‐IVb whole‐genome sequences (11,083 NT) (Table 2), plus an additional four from GenBank (GQ385941, KY359355–KY359357), totaling 44 isolates.

We constructed phylogenetic trees of haplotypes using maximum likelihood in PHYML and Bayesian analysis with MRBAYES v3.2.1 (Ronquist & Huelsenbeck, 2003), to evaluate evolutionary changes and relationships. Comparative divergence times were estimated using BEAST v1.10.4 (Suchard et al., 2018), with JMODELTEST output, a relaxed molecular clock having lognormal distribution, and sampling every 50,000 of 500,000,000 generations. Outputs were assessed with TRACER v1.5 (in BEAST) to ensure stationarity. Collection dates were used as calibration points, and tree branches set following PHYML output. Numbers of nucleotide substitutions per site per year (*k* = substitutions site^−1^ yr^−1^) were determined from pairwise (p) distances. Nucleotide (NT) and amino acid (AA) substitutions were evaluated for all isolates and compared to MI03GL (haplotype “a”).

Population genetic relationships using the *G*‐gene sequence data were tested among the following: (a) three time periods (Early: 2003–2006, Middle: 2007–2010, and Later: 2011–2017 [with 2018–2020 having no reported incidents]), (b) three primary geographic regions (Upper Great Lakes: Lake Superior, Lake Michigan, Lake Huron, Budd Lake, Lake Simcoe, and inland Wisconsin lakes; Central Great Lakes: Lakes Erie and St. Clair, Baseline Lake; and Lower Great Lakes: Lake Ontario, St. Lawrence River, and New York Finger Lakes), and (c) the top six fish species affected (freshwater drum, gizzard shad, largemouth bass, smallmouth bass [*Micropterus dolomieu*], round goby, and yellow perch). Time periods were formulated to contain comparable numbers of samples in each of the three groups. Three isolates from Clearfork Reservoir, Ohio, were excluded from population analyses due to their location outside of the Great Lakes drainage, and small sample size. We further compared populations from individual water bodies (Lake Michigan, Lake Huron, Budd Lake, Lake St. Clair, Lake Erie, New York Finger Lakes, and St. Lawrence River), excluding Lake Superior since it comprised a single isolate at a single time point.

We calculated haplotypic and nucleotide diversity, and number and relative proportion of private haplotypes in populations with ARLEQUINv3.5 (Excoffier & Lischer, 2010). Evolutionary relationships among haplotypes are depicted with POPART (https://popart.otago.ac.nz) and TCS networks (Clement, Posada, & Crandall, 2000). Pairwise divergences between populations were analyzed using *θ*_ST_ (*F*_ST_ analogue; Weir & Cockerham, 1984) in ARLEQUIN and with exact tests of differentiation (*χ* ^2^) in GENEPOP v4.6 (Raymond & Rousset, 1995; Rousset, 2008). The latter employed a MCMC procedure with 1,000 batches and 10,000 iterations to randomly sample allelic frequencies. Probabilities were adjusted with sequential Bonferroni correction (Rice, 1989) and reported both prior and after adjustment, to identify borderline cases.

Tajima's (1989) *D* tests in ARLEQUIN evaluated possible influence of selection. We also further examined selection pressures using unconstrained Bayesian approximation (FUBAR) (Murrell et al., 2013) to identify positive or purifying selection. Since FUBAR's assumption of constant selection might not accurately represent IVb, we also used MEME (mixed‐effects model of evolution), which can detect positive selection, under strong purifying selection or the removal of detrimental variants (Murrell et al., 2013). FUBAR and MEME were run with HyPHY on DataMonkey (www.datamonkey.org), with significance evaluated using posterior probability >.95 for FUBAR and *p* < .05 for MEME.

Analysis of molecular variance (AMOVA) in ARLEQUIN assessed hierarchical partitioning of genetic variation among the following: (a) three geographic regions (Upper, Middle, and Lower Great Lakes) and their sampling events, and (b) three time periods (Early, Middle, and Later) and their component sampling events. We tested these scenarios for all NT substitutions and separately for amino acid (AA) substitutions, using the *G*‐gene.

A neighbor‐joining genetic distance tree analyzed population relationships using Reynolds' *R*_ST_ genetic distances (Reynolds, Weir, & Cockerham, 1983) in PHYMLv3.697 (Felsenstein, 2007) with 10,000 bootstrap pseudoreplications (Felsenstein, 1985). Possible relationships between genetic distance (*θ*_ST_) and geographic distance were evaluated with separate Mantel (1967) tests for the Early, Middle, and Later time periods, using shortest waterway distances (km) between outbreak locations or the most direct road route for landlocked locations. We also tested the relationship between genetic distance (using all samples) and time (sampling years).

## 3 RESULTS

### 3.1 VHSV‐IVb detections

We collected and analyzed 2,649 individuals from 45 fish species in 2015–2017, among which our qPCR assay results identified just 21 VHSV positives (0.82%). Only two positives were found in 2015—from a white perch (*Morone americana*) and a round goby in Lake Erie's central basin. Each of those possessed a unique *G*‐gene haplotype—“u” and “v” (Table 1). In 2016, 19 individuals tested positive: 14 from Lake Erie, including one from the central basin (largemouth bass), and 13 from a single sampling event in the western basin (one emerald shiner [*Notropis atherinoides*], nine gizzard shad, one pumpkinseed [*Lepomis gibbosus*], and two largemouth bass), and five from Lake Michigan (four round goby and one alewife [*Alosa pseudoharengus*]). These had two new haplotypes: 13 with “w” and six with “x” (Table 1). No VHSV positives were detected from 1,003 dreissenid mussels collected in May–September 2015; thus, no further testing was conducted. Additionally, none of the 2017 fishes we sampled tested VHSV positive.

**FIGURE 3:**
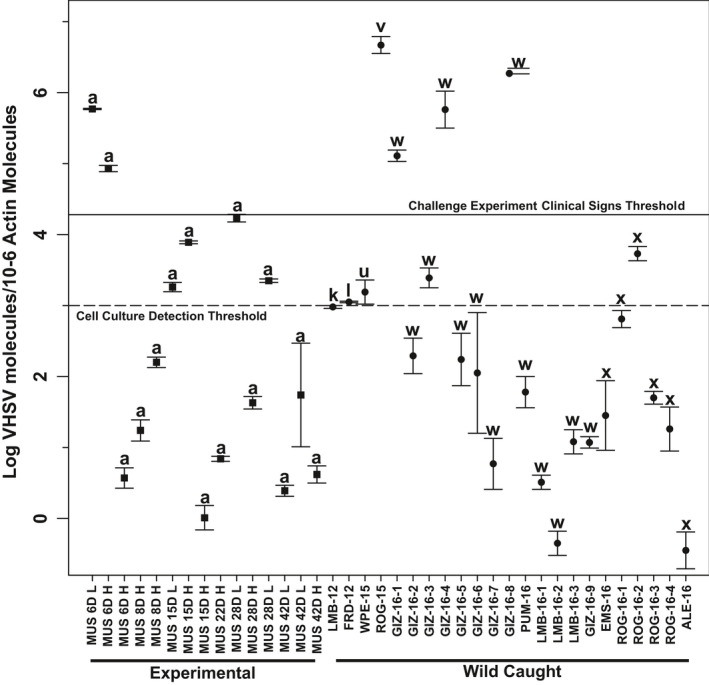
Concentrations of VHSV‐IVb (±standard error) in wild‐caught fish tissues, compared to results from experimental laboratory haplotype “a” challenged muskellunge, determined with qPCR assay developed in Stepien's laboratory using internal standards (Pierce, Willey, Palsule, et al., 2013). Laboratory samples (squares) are named by the number of days (6‐42D) after VHSV‐IVb inoculation, H: high virus dosage (1 × 10^5^ pfu/ml), and L: low dosage (100 pfu/ml) (data from Pierce, 2013). Haplotype of each sample is listed above its standard error bars. Solid line denotes the experimental threshold for clinical signs of disease and dashed line the cell culture detection threshold (Pierce, Willey, Palsule, et al., 2013). Wild‐caught samples (circles) are designated by abbreviated common name, followed by collection year and sample number (Table 1). Fish species names: ALE, alewife; EMS, emerald shiner; FRD, freshwater drum; GIZ, gizzard shad; LMB, largemouth bass; MUS, muskellunge; PUM, pumpkinseed; ROG, round goby; WPE, white perch

Our qPCR analyses of field‐collected fishes (Figure 3) discerned VHSV‐IVb concentrations (log values) for the 2015 white perch (haplotype “u”) of 1.8 × 10^3^ VHSV/10^6^ β‐actin molecules, with the round goby 2015 individual (“v”) being much higher at 5.2 × 10^6^. The latter concentration was the highest recorded, much above the challenge experiment threshold for clinical signs of disease based on haplotype “a,” yet the fish exhibited no visible VHSV signs (Pierce, Willey, Palsule, et al., 2013). A wide range of viral concentrations (5.1 × 10^1^ to 1.9 × 10^6^) occurred in our 2016 field‐collected positives (“w” and “x”), as had been found in the laboratory‐challenged fish (haplotype “a”) experiments (the latter from Pierce, 2013). Three 2016 gizzard shad individuals from Lake Erie possessing VHSV‐IVb haplotype “w” also had virus concentrations that were above the clinical disease sign threshold (Figure 3).

### 3.2 Evolutionary patterns

**FIGURE 4:**
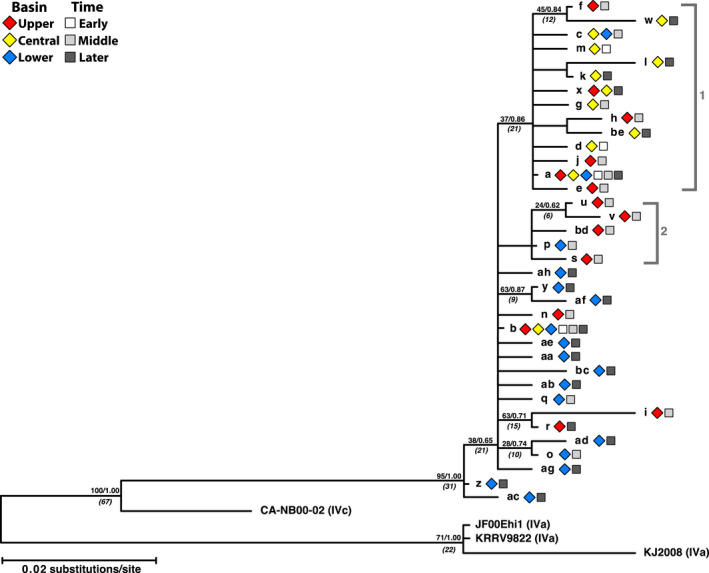
VHSV‐IVb *G*‐gene phylogeny. Phylogenetic tree of VHSV haplotypes based on the *G*‐gene from maximum likelihood and Bayesian analyses. Values above nodes = 2,000 bootstrap pseudoreplicates/Bayesian posterior probabilities. Values in parentheses and italics = estimated divergence time (years) (Stepien et al., 2015). VHSV‐IVa (AB179621) served as the out‐group. Two clades discussed here (1 and 2) are designated by brackets. Symbols designate area in the Great Lakes (diamonds) and time period (squares)

We analyzed a total of 184 *G*‐gene sequences comprising 36 haplotypes, and a subset of those for whole‐genome sequences containing 34 haplotypes. Figure 4 shows the consensus *G*‐gene phylogenetic tree from maximum likelihood (PHYML) and Bayesian (MR. BAYES) analyses, which was rooted to genogroup VHSV‐IVa. Estimated divergence times are indicated (BEAST). This tree is congruent with the tree from Stepien et al. (2015), but expanded to include the additional haplotypes to date. There are two major clades (labeled 1 and 2), with the new 2016 and 2017 haplotypes (“w,” “x,” and “bd”) contained inside clade 1, along with the original haplotype “a.” The new haplotypes from 2015 (“u” and “v”) are in clade 2. Evolutionary rates for the *G*‐gene were similar in both datasets (partial *G‐*gene: 1.00 × 10^−4^ substitutions site^−1^ yr^−1^; whole *G*‐gene: 8.51 × 10^−5^). The overall rate for the entire genome dataset was slower, at 6.64 × 10^−5^ (Table 3).

**TABLE 3:** Single‐nucleotide polymorphisms (SNPs) from each gene's coding region and the combined noncoding regions (NCDS) in VHSV‐IVb sequence variants, across the whole genome

Region	Length NT (AA)	*N* changes NT (AA)	% changes NT (AA)	dN/dS	*N* Tv	Tv/Ts	Evolutionary rate
*N*‐gene	1,215 (405)	28 (10)	0.023 (0.025)	0.357	7	0.250	5.83E‐05
*P*‐gene	669 (223)	13 (3)	0.019 (0.014)	0.231	2	0.154	7.18E‐05
*M‐*gene	606 (202)	17 (9)	0.028 (0.045)	0.529	2	0.118	7.93E‐05
*G*‐gene	1,524 (508)	37 (18)	0.024 (0.035)	0.486	6	0.162	8.51E‐05
*Nv*‐gene	369 (123)	14 (7)	0.038 (0.057)	0.500	4	0.286	9.76E‐05
*L*‐gene	5,955 (1,985)	112 (38)	0.019 (0.019)	0.339	18	0.161	5.02E‐05
NCDS	745 (N/A)	32 (N/A)	0.043 (N/A)	N/A	3	0.094	1.40E‐04
Total	11,083 (3,446)	253 (85)	0.023 (0.025)	0.166	42	0.166	6.64E‐05

Note

The number of nucleotides (NT) is reported in front of the number of amino acid (AA) changes (the latter are in parentheses). The proportion of nonsynonymous (dN) to synonymous (dS) changes, number of transversions (Tv), and the proportion of transversions to transitions (Ts) are given, along with the evolutionary rate. Totals are in the final row.

**FIGURE 5:**
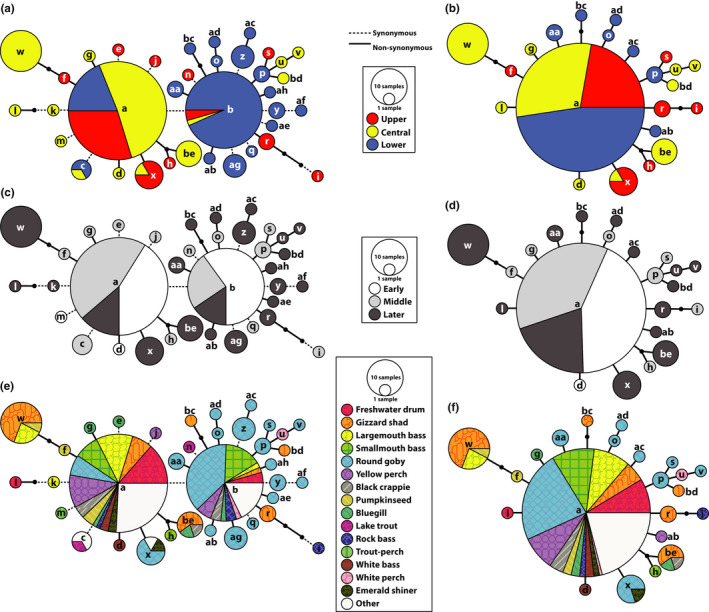
*G*‐gene haplotype networks. Partial *G*‐gene sequences (669 NT) from 176 isolates using POPART (https://popart.otago.ac.nz) and TCS (Clement et al., 2000) for (a, b) Great Lakes regions (Upper, Central, Lower), (c, d) time periods (Early, Middle, Later), and (e, f) host species. a, c, and e are based on nucleotide substitutions and b, d, and f on amino acid changes. Circles are sized according to frequency of the haplotype in the population. Lines denote a single substitution step between haplotypes, with dashed lines for synonymous changes and solid lines for nonsynonymous changes. Small, unlabeled black circles represent hypothesized haplotypes. The “Other” category in e–f contains all host species, in which three or fewer nonunique isolates were detected: alewife (*Alosa pseudoharengus*), amphipod (*Diporeia* spp.), brown bullhead (*Ameiurus nebulosus*), burbot (*Lota lota*), channel catfish (*Ictalurus punctatus*), Chinook catfish (*Oncorhynchus tshawytscha*), common carp (*Cyprinus carpio*), cisco (*Coregonus artedi*), lake whitefish (*C. clupeaformis*), leech (*Myzobdella lugubris*), muskellunge (*Esox masquinongy*), northern pike (*E. lucius*), rainbow trout (*Oncorhynchus mykiss*), sea lamprey (*Petromyzon marinus*), shorthead redhorse (*Moxostoma macrolepidotum*), and walleye (*Sander vitreus*)

Figure 5 depicts the *G*‐gene haplotype networks, including separate networks (b, d, f) based on nonsynonymous substitutions alone (those with AA changes). Two predominant haplotypes, “a” and “b,” are centrally located as the largest circles, containing 74 (40%) and 45 (24%) respective isolates. One single synonymous transition from cytosine to guanine at position 3,996 separated haplotypes “a” and “b.” Thirteen unique haplotypes descend from “a,” with mean divergence of 1.46 ± 0.22 NT. Twenty‐one unique haplotype descendants surround “b,” diverging by a mean of 1.67 ± 0.19 NT. Regional patterns are apparent (Figure 5a, Table 4), with 81% of haplotype “a” occurrences in the Upper (30%) and Central Great Lakes (51%), and 93% of “b” in the Lower Great Lakes. Similar geographic separations characterized the descendants: the “a” group was more prevalent in the Upper (*N* = 9, 25%) and Central Great Lakes (*N* = 24, 67%) and “b” in the Lower Great Lakes (*N* = 23, 74%).

**TABLE 4:** *G*‐gene haplotype numbers, diversity, and private haplotypes per time period (A) and for Great Lakes regions (B)

A										
	*N* haplotypes (Total Isolates)	*N* haplotypes “a” Group (%)	*N* haplotypes “b” group (%)	Haplotypic diversity ± *SE*	Mean nucleotide diversity ± *SE*	*N* private haplotypes (%)	Proportion private haplotypes	*N* haplotypes Upper Great Lakes (total)	*N* haplotypes Central Great Lakes (total)	*N* haplotypes Lower Great Lakes (total)
Early period	4 (58)	3 (75)	1 (25)	0.540 ± 0.026	0.00086 ± 0.00078	2 (50%)	0.034	1 (7)	3 (22)	2 (29)
Middle period	14 (58)	7 (50)	7 (50)	0.672 ± 0.061	0.00169 ± 0.00124	12 (86%)	0.276	9 (18)	4 (15)	7 (25)
Later period	22 (68)	6 (27)	16 (72)	0.915 ± 0.017	0.00450 ± 0.00263	20 (91%)	0.735	3 (17)	8 (24)	13 (27)
Total	36 (184)	14 (39)	22 (61)	0.778 ± 0.024	0.002671 ± 0.00172	34 (94%)	0.370	11 (42)	14 (61)	17 (81)

B										
	*N* haplotypes (total isolates)	*N* haplotypes “a” group (%)	*N* haplotypes “b” group (%)	Haplotypic diversity ± *SE*	Mean nucleotide diversity ± *SE*	*N* private haplotypes (%)	Proportion private haplotypes	*N* haplotypes early period (total)	*N* haplotypes middle period (total)	*N* haplotypes later period (total)
Upper Great Lakes	11 (42)	6 (55)	5 (45)	0.642 ± 0.084	0.00186 ± 0.00133	8 (73)	0.214	1 (7)	9 (18)	3 (17)
Central Great Lakes	14 (61)	8 (57)	6 (43)	0.661 ± 0.057	0.00260 ± 0.00170	10 (71)	0.426	3 (22)	4 (15)	8 (24)
Lower Great Lakes	17 (81)	2 (11)	16 (89)	0.704 ± 0.045	0.00153 ± 0.00115	14 (82)	0.284	2 (29)	7 (25)	13 (27)
Total	36 (184)	14 (39)	22 (61)	0.778 ± 0.024	0.002671 ± 0.00172	32 (89)	0.321	4 (58)	14 (58)	22 (68)

Temporal patterns also are apparent in the networks (Figure 5c, Table 4). Haplotypes “a” and “b” were most abundant during the Early time period, comprising 51% and 46% of the samples. During the Middle time period, “a” remained more common (56%) than “b” (19%), and both declined during the Later period (16% and 10%), disappearing after 2011. Most haplotypes from the Later time period are genetically distant from the central “a” and “b” haplotypes (mean = 2.45 ± 0.17 steps), with all five from 2015 to 2017 being unique. None of the “b” descendent haplotypes originated during the Early time period, with six (29%) appearing during the Middle, and the remaining 15 (71%) during the Later period.

A diversity of host species (Figure 4e–f) possessed either “a” or “b” haplotypes; none predominated in “a,” but haplotype “b,” and its descendants frequently were found in round goby (constituting 38% of the occurrences of “b”). Overall, “a” was in 13 host species and “b” in 11, with nine having either “a” or “b.” Multiple occurrences of “a” group haplotypes were in gizzard shad (*N = *12, including haplotype “w”), round goby (*N* = 4, all “x”), and bluegill (*Lepomis macrochirus*, *N* = 3) (Figure 5e). All emerald shiner and white bass (*Morone chrysops*) contained haplotype “a,” and the single white perch with VHSV possessed “b.” Both of the positive invertebrate samples (leech and amphipod) had “a” (Faisal & Schulz, 2009; Faisal & Winters, 2011).

Mean number of substitutions and relative percentage of AA changes in VHSV‐IV significantly increased over time (Table 5A). The Later time period contained the most, averaging 1.62 substitutions and 1.05 (65%) AA changes. The Middle period averaged 1.39 and 0.85 (61%), and the Early period had just 1.00 and 0.33 (33%). Among the three geographic regions, the Central Great Lakes possessed the most, having 1.69 mean NT and 1.23 (72.8%) AA changes, with fewer in the Upper (1.50, 1.10; 73.3%) and Lower Great Lakes (1.25, 0.50; 40%); that is, more substitutions were synonymous in the latter.

**TABLE 5:** Mean numbers of nucleotide (NT) and amino acid (AA) changes (± standard errors) among VHSV‐IVb haplotypes for (A) time periods and (B) geographic regions for the *G*‐gene

A. Time period				
	Early	Middle	Later	Total
*G*‐NT	1.000 ± 0.000	1.385 ± 0.241	1.619 ± 0.161	4.004 ± 0.402
*G*‐AA	0.333 ± 0.333	0.846 ± 0.274	1.048 ± 0.223	2.227 ± 0.830

B. Region				
	Upper	Central	Lower	Total
*G*‐NT	1.500 ± 0.307	1.692 ± 0.237	1.250 ± 0.112	4.442 ± 0.656
*G*‐AA	1.100 ± 0.314	1.231 ± 0.303	0.500 ± 0.183	2.831 ± 0.800

### 3.3 Congruence with whole‐genome analyses

Lower sample sizes (*N* = 45) and lack of available samples from some areas and temporal periods precluded more in‐depth population analyses using whole‐genome sequences, versus the larger sample sizes available for the *G*‐gene alone (see Table 3).

**FIGURE 6:**
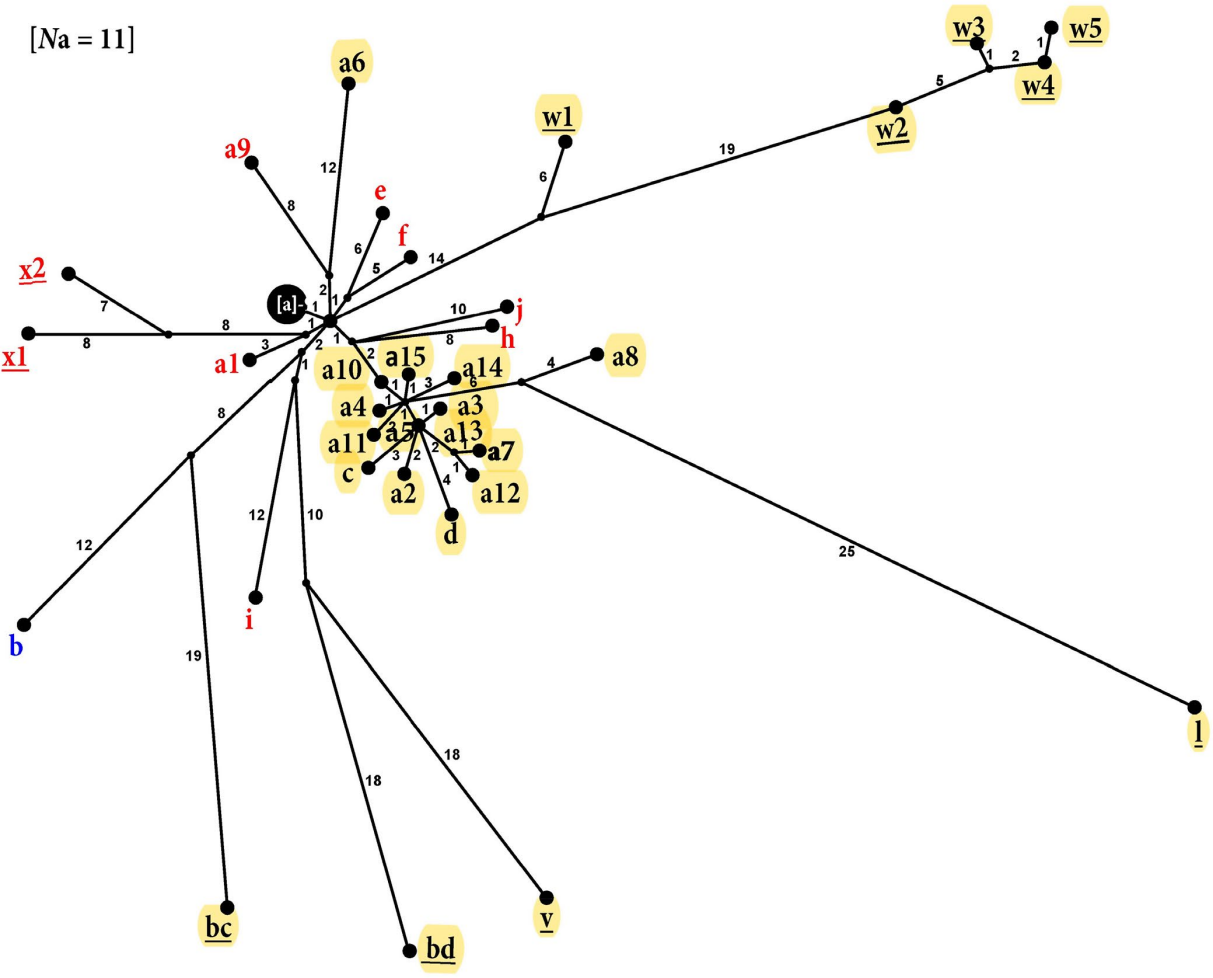
Whole‐genome haplotype network. Gene sequences (11,083 NT) from 44 isolates having 34 haplotypes (designated in Table 2) illustrated with POPART and TCS. Numbers on branches denote numbers of NT changes. Red = Upper Great Lakes, Yellow = Central Great Lakes, Blue = Lower Great Lakes, and Underline = Haplotypes from the Later time period. Individual isolates sharing haplotype “a” for the whole‐genome sequences were the following: E06WBc, C06NP, C06RB, C06SR, C06YP, C06FB, M08AMa‐b, C08LEa‐b, and C09MU, along with C03MU (see Table 2)

In the whole‐genome analyses (Figure 6), the original haplotype “a” (sequence GQ385941, from the original IVb 2003 muskellunge isolate MI03GL) characterized 11 other isolates, including one in 2006 from Lake Erie in the Central Great Lakes, eight from Lake St. Clair in the Central Great Lakes (five in 2006 and one in 2009), along with the 2008 samples of invertebrates that were positive from Lakes Michigan and St. Clair. All of these identical whole‐genome haplotype “a” individuals were from either the Early (86%) or the Middle time periods (14%). In whole genome analysis, haplotype “b” diverged by 20 NT steps from “a,” having a sole representative in the Upper Lakes during the Middle time period (note that we lacked more isolates to analyze from the Lower Lakes). The whole genome network shows a pronounced star‐like cluster of 13 “a”‐derived haplotypes in Lake Erie (labeled a2–5, a7, a10–15, c, and d), which diversified from haplotype “a” during 2006–2008. The “w” isolates and its variants (all from 2016 in Lake Erie) are the most divergent, differing from “a” by 20–41 NT substitutions. Haplotypes “l” from 2012 and “v” from 2015 also are respectively distant, showing unique trajectories (Figure 6). Overall relationships among geographic locations and across time are congruent between the whole‐genome and *G‐*gene networks.

### 3.4 Population genetic patterns

Haplotype network results were statistically supported by pairwise genetic divergence (*θ*_ST_) analyses. Pronounced population genetic divergence occurred over time, with the Later period differing from both the Early and Middle time periods (Table 6A). Populations from all three regions (Upper, Central, and Lower Great Lakes) significantly diverged (Table 6B), with the greatest distinction between the Central and Lower Lakes. Among individual water bodies (Table 6C), the St. Lawrence River differed the most. Virus populations from Lakes Erie and St. Clair were more similar to each other, but very divergent from those in Lakes Michigan and Ontario.

**TABLE 6:** Pairwise genetic divergences of VHSV populations between (A) sampling time periods, Early (2003–2006), Middle (2007–2010), and Later (2011–2018), (B) Great Lakes regions (Upper, Central, Lower), and (C) individual water bodies, based on variation for the (1) *G*‐gene and (2) entire genome data sets, using exact tests (GENEPOP; above diagonal) and *θ*_ST_ divergences (ARLEQUIN; below diagonal)

A1			
	Early *N* = 58	Middle *N* = 57	Later *N* = 65
Early	—	NS	*
Middle	0.019	—	*
Later	0.153*	0.123*	—

A2			
	Early *N* = 18	Middle *N* = 15	Later *N* = 12
Early	—	NS	*
Middle	0.001	—	*
Later	0.218*	0.197*	—

B2		
	Upper *N* = 9	Central *N* = 34
Upper	—	*
Central	0.111*	—

B1			
	Upper *N* = 39	Central *N* = 61	Lower *N* = 80
Upper	—	*	*
Central	0.111*	—	*
Lower	0.264*	0.348*	—

C1								
	Lake Michigan *N* = 19	Budd Lake *N* = 12	Lake Huron *N* = 7	Lake St Clair *N* = 19	Lake Erie *N* = 43	Lake Ontario *N* = 21	St. Lawrence River *N* = 52	Finger Lakes *N* = 6
Lake Michigan	—	*	NS	*	*	*	*	NS
Budd Lake	0.072*	—	*	NS	*	*	*	NS
Lake Huron	0.037	0.198*	—	*	NS	NS	*	NS
Lake St. Clair	0.136*	0.135	0.180*	—	*	*	*	NS
Lake Erie	0.165*	0.119*	0.115	0.114*	—	*	*	NS
Lake Ontario	0.095*	0.220*	0.001	0.230*	0.189*	—	*	NS
St. Lawrence River	0.393*	0.561*	0.257*	0.563*	0.447*	0.207*	—	*
Finger Lakes	0.001	0.153	0.001	0.180	0.113	0.001	0.318*	—

C2			
	L. Erie and L.Ontario/SLR *N* = 24	L. St Clair *N* = 10	L. Michigan and Budd L. *N* = 9
L. Erie and L. Ontario/St. Lawrence R.	—	*	*
L. St Clair	0.111*	—	*
L. Michigan and Budd L.	0.348*	0.264	—

* *p* ≤ .05, **remained significant (*P* < α) following sequential Bonferroni correction, NS = *p* > .05

Hierarchical relationships among the major population groups using AMOVA (Table 7A) revealed significant partitioning among the three Great Lakes' regions (18%, *p* < .001) and among their sampling time periods (51%, *p* < .001), totaling 69% of the variation. AMOVA (Table 7B) found less but significant variation when variation first was partitioned among the three time periods (0.52%, *p* < .001), and more among their component sampling events (67%, *p* < .001). Analyses for AA substitutions (Table 7B) indicated greater genetic structuring among the time periods (7%, *p* < .001) and their sampling events (63%, *p* < .001) than among geographic regions (Table 7B).

**TABLE 7:** Relative distribution of genetic variation among VHSV‐IVb isolates using analysis of molecular variance (AMOVA, Excoffier, Smouse, & Quattro, 1992), calculated from partial *G‐*gene sequences for (A) nucleotide sequences, and (B) amino acid changes, using ARLEQUINv3.5.1.3 (Excoffier & Lischer, 2010)

Distribution of genetic variation	% Variation	*Φ*	*p*
A. *G*‐gene nucleotide substitutions			
1. a. Among three Great Lakes Regions (Upper, Central, Lower)	17.99	0.624*	<.001*
1. b. Sampling events within the three regions	51.14	0.691*	<.001*
1. c. Within samples	30.87	0.180*	.016*
2. a. Among time periods (Early, Middle, Later)	0.52	0.671*	<.001*
2. b. Sampling events within the three time periods	66.79	0.673*	<.001*
2. c. Within samples	32.69	0.005	.395
B. *G*‐gene Amino Acid Substitutions			
1. a. Among three Great Lakes Regions (Upper, Central, Lower)	0.100	0.698*	<.001*
b. Sampling events within the three regions	69.77	0.699*	<.001*
1. c. Within samples	30.13	0.001	.304
2. a. Among time periods (Early, Middle, Later)	7.17	0.682*	<.001*
2. b. Sampling events within the three time periods	63.31	0.705*	<.001*
2. c. Within samples	29.51	0.072	.131

* Significant.

**FIGURE 7:**
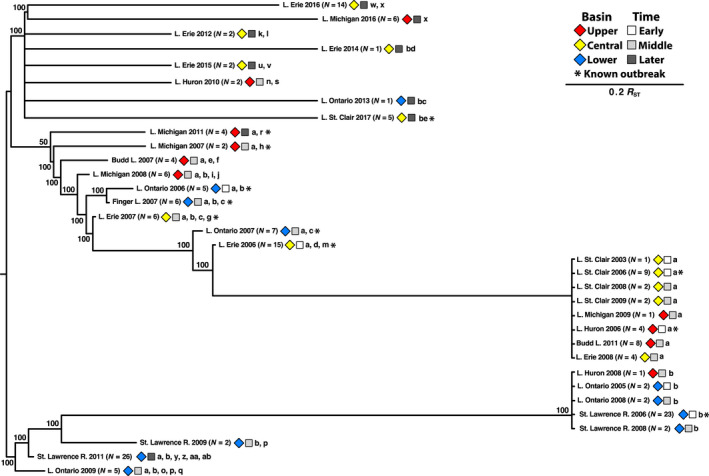
Neighbor‐joining genetic distance tree depicting relationships among VHSV‐IVb population samples. Reynolds' (1983) genetic distances (*R*_ST_) used on *G*‐gene haplotypes and their frequencies in PHYLIP (Felsenstein, 2007). Bootstrap percentage support for nodes from 10,000 replications is shown. Sample sizes (*N*) are in parentheses. Symbols designate area in the Great Lakes (diamonds) and time period (squares). *Samples from confirmed fish kill/outbreak events

The neighbor‐joining genetic distance tree (Figure 7) shows the relationships among VHSV‐IVb populations, which were each collected from a single area at a single time. The tree depicts two primary population clusters, whose upper cluster is dominated by “a” and its descendants, and lower cluster by haplotype “b” and its descendants. Lake St. Clair samples from 2003 to 2009 cluster together, along with others from the Upper and Central Great Lakes. The more recent sample occurrences from Lake St. Clair (2017) appear very distant from those. Lake Michigan and Budd Lake samples are spread apart across the “a” cluster. The lower cluster mostly contains samples from the Lower Great Lakes region. No trends are apparent among outbreak events (* on Figure 7).

**FIGURE 8:**
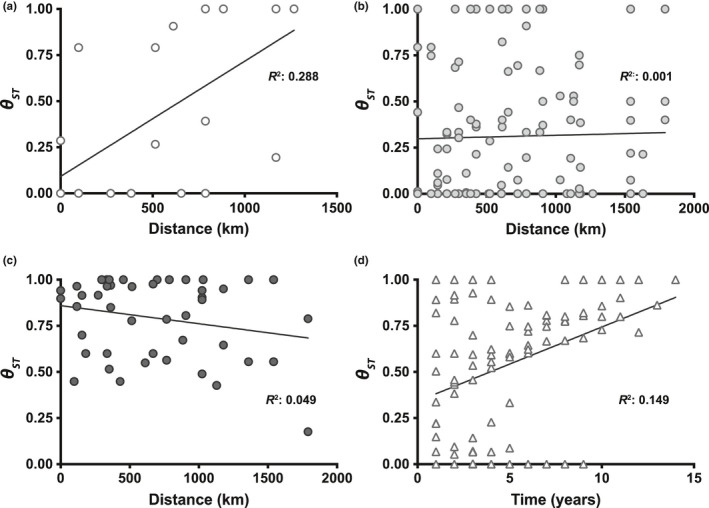
Tests for significant relationship between genetic divergence (*θ*_ST_) among VHSV *G‐*gene sampling groups versus geographic distance (a–c, nearest waterway distance, km) or time (d, years). (a) Early time period (*y* = 0.001*x* + 0.091, *R* ^2^ = .288, *p* = .006*), (b) Middle time period (*y* = 1.86e‐5*x* + 0.298, *R* ^2^ = .001, *p* = .278; N.S.), (c) Later time period (*y* = −9.82e‐5*x* + 0.860, *R* ^2^ = .049, *p* = .709; N.S.), and (d) all samples (*y* = 0.040*x* + 0.342, *R* ^2^ = .149, *p* = .002*). N.S., not significant, * = significant

Mantel tests (Figure 8) support a positive relationship between genetic distance and geographic distance for the Early time period (Figure 8a; *p* = .006), but not for the Middle (Figure 8b) or Later (Figure 8c) periods. The Early period samples had just four unique haplotypes (*N = *58), whose population distribution showed increasing genetic divergence (*θ*_ST_) with increasing geographic distance from the original haplotype “a's” location in Lake St. Clair. The relationship between genetic divergence (*θ*_ST_) and time (years) was significant across the entire dataset (*R* ^2^ = .15, *p* = .002; Figure 8d). Frequency of identical haplotypes fell off sharply after the 2005–2007 outbreaks, with most isolates from 2009 and beyond having new and divergent haplotypes, when “a” and “b” became rare (disappearing after 2011). Although the Middle and Later periods do not display correlation between *θ*_ST_ and geographic distance (Figure 7b,c), this likely reflects overall increasing diversity of haplotypes.

Tajima's *D* tests indicate that the *G*‐gene and the complete genome have been under significant purifying selection, possessing negative values (Table 8). Only the population from the Middle time period was significant, with the Early and Later time periods also being negative (but not significant). The Upper and Lower Great Lakes populations likewise were significant. For tests on individual water bodies, the St. Lawrence River population alone was significant. The top eight species also were analyzed for selection pressure, with round goby being significant (Table 8). These variations likely reflect sample size effects, since far more round goby individuals (*N* = 50) were collected than any other individual species (*N* = 5–23).

**TABLE 8:** Tajima's *D* test values (ARLEQUIN) for selection pressures on VHSV‐IVb evolution based on (A) all samples, (B) geographic region (Upper, Central, Lower Great Lakes), (C) time period (Early [2003–2006], Middle [2007–2010], Later [2011–2018]), (D) individual water bodies, and (E) host species

	Dataset	Test group	Sample size	Tajima's *D*	*p*
A. Haplotypes	1. *G*‐gene	All samples	186	−2.008*	.002*
	2. Whole genome	All samples	044	−2.412*	.000*
B. Regions	1. *G*‐gene	Upper Great Lakes	039	−1.982*	.007*
		Central Great Lakes	064	−1.059	.145
		Lower Great Lakes	082	−1.957*	.005*
	2. Whole genome	Central and Lower Great Lakes	038	−2.031*	.006*
		Upper Great Lakes	009	−1.672*	.027*
C. Time	1. *G*‐gene	Early Period	059	−0.225	.439
		Middle Period	057	−2.042*	.004*
		Later Period	068	−1.063	.139
	2. Whole genome	Early Period	018	−2.369*	.001*
		Middle Period	015	−2.127*	.003*
		Later Period	012	−1.009	.154
D. Water bodies	1. *G*‐gene	Lake Michigan	019	−1.223	.108
		Budd Lake	012	−1.451	.066
		Lake Huron	007	−0.598	.332
		Lake St. Clair	020	0.939	.835
		Lake Erie	044	−0.989	.169
		Lake Ontario	022	−1.102	.153
		St. Lawrence River	053	−2.099*	.002*
		Finger Lakes	006	−0.050	.443
E. Species	1. *G*‐gene	Black crappie	005	1.225	.943
		Freshwater drum	013	−0.909	.223
		Gizzard shad	023	0.124	.594
		Pumpkinseed	005	−0.175	.437
		Bluegill	006	−1.011	.207
		Round goby	050	−1.711*	.022*
		Largemouth bass	014	−0.908	.201
		Smallmouth bass	014	−0.387	.359
		Yellow perch	012	−0.382	.313
		Other	049	−1.946*	.007*

* *p* ≤ .05

Further analyses to uncover possible selection using FUBAR and MEME on the entire genome data set also indicated that purifying selection characterized IVb's *N‐* (codon 313), *G‐* (codon 342), and *L‐*genes (six codons: 8, 119, 333, 460, 1,284, and 1,758) (Table 9). One codon (*L*, 1,758) indicated purifying selection, and three codons implied diversifying selection—*G* (103, 431) and *Nv* (25). MEME implicated diversifying selection at *G*431.

**TABLE 9:** Positive (diversifying) or negative (purifying) selection pressures on individual codons determined by FUBAR (fast, unconstrained Bayesian approximation) and MEME (mixed‐effects model of evolution) analyses (Murrell et al., 2013) for VHSV‐IVb

Gene	FUBAR diversifying (*pp* > .95)	FUBAR purifying (*pp* > .95)	MEME diversifying (*p* < .05)
*N*	0	1 (313)	0
*P*	“ “	0	“ “
*M*	“ “	“ “	“ “
*G*	2 (103, 431)	1 (342)	1 (431)
*Nv*	1 (25)	0	0
*L*	0	6 (8, 119, 333, 460, 1,284, 1,758)	“ “

Abbreviation: *pp*, posterior probability. “ “ = same as above.

## 4 DISCUSSION

### 4.1 VHSV‐IV occurrences and evolutionary trajectory

VHSV‐IVb has undergone extensive evolutionary changes across its less than two decades in the Great Lakes, showing significant population spatial and temporal patterning, and increased genetic differentiation. Its haplotype distributions showed geographic structuring among the Upper, Central, and Lower Great Lakes. Amino acid changes also revealed significant diversification over time. Such increased genotypic and phenotypic variation may allow a virus to overcome its hosts' immune systems, both within and among host species (Novella & Presloid, 2012; Stepien et al., 2015). This diversity may allow the virus population to persist under consistent and/or variable environmental conditions, and to enter new hosts and their habitats.

VHSV‐IVb continued to diversify following a quasispecies pattern, radiating new variants from the original central “a” and “b” haplotypes. Ojosnegros and Beerenwinkel (2010) postulated that viral variants displaying high virulence frequently are outcompeted over time by less virulent ones, which can better persist in the host population. Our 2015–2016 sampling uncovered just a few fishes that were positive for the virus, which contained new haplotypes and encompassed a wide realm of viral concentrations. Those fish hosts all lacked hemorrhages and other clinical signs of disease. In contrast, the first VHSV‐IVb outbreaks were characterized by high virulence, hemorrhaging, and mass fish die‐offs (Kim & Faisal, 2011). Similarly, the classic example of Australian Myxoma Virus began with high mortality in feral rabbits, which lessened over time due to coadaptations between the virus and host populations (Alves et al., 2019; Elsworth et al., 2014). That pattern also appears to have characterized VHSV‐IVb, whose initial host fish populations may have been more susceptible than later ones, followed by increased acclimation and resistance of the hosts. Meanwhile, the virus continued to differentiate over time.

### 4.2 Evolutionary patterns across space and time

Evolutionary diversification of VHSV‐IVb, based on our *G*‐gene results, radiated from the original haplotypes “a” and “b,” with the “a” descendent group primarily found in the Upper and Central Great Lakes, and the “b” group in the Lower Great Lakes and St. Lawrence River. The two original haplotypes may have originated from separate introductions into the Great Lakes, with “a” in the Upper and Central Great Lakes, and “b” in the Lower Great Lakes. An alternate hypothesis is that “b” descended from “a,” with the latter first appearing in Lake St. Clair ca. 2003. VHSV‐IVb haplotypes “a” and “b” appeared nearly monotypic in the host populations during the Early time period (2003–2006), accounting for 90% of the known isolates, but both haplotypes disappeared after 2011.

Our results revealed significant genetic divergences among VHSV‐IVb population groups from the Upper, Central, and Lower Great Lakes, with greatest difference between those in the Central and Lower Great Lakes. Of the latter, the St. Lawrence River population was the most divergent. Populations in Lakes Erie and Ontario also significantly differed from most others. Our results indicate that most sequence diversification and new haplotypes occurred during the Middle and Later time periods, that is, after the major 2005 and 2006 outbreaks.

The closely related IHNV (=*Salmonid novirhabdovirus*) also exhibited geographic patterning among three distinct geographic Northeastern Pacific regions, with its “M” group having more substitutions than its “L” and “U” groups (Kurath et al., 2003). In 1985, IHNV was reported in China, where it likely was introduced from Japan, and now constitutes a distinct clade (Xu et al., 2018). Since regional differences in VHSV‐IVb are based on a history of less than two decades in the Great Lakes, it appears likely that geographic divergence and genetic diversity may develop further (unless the virus “disappears”).

In our study, genetic divergence (*θ*_ST_) was strongly correlated with geographic distance among samples. The relationship between genetic divergence and time (years) also showed significant positive correlation, indicating continued changes over time. Increased genetic divergence was accompanied by greater overall population variability, consistent with the quasispecies theory.

In contrast with the patterns we discerned for VHSV‐IVb, evolutionary relatedness of IHNV *G*‐gene sequences along the Northeastern Pacific did not correspond to changes over time (Kurath et al., 2003). Another Northeastern Pacific IHNV study identified greater diversity in viral isolates in later years, but attributed those to increased sampling effort and surveillance (Black, Breyta, Bedford, & Kurath, 2016), which was not the case in our study.

Like genetic structuring in VHSV‐IVb and IHNV, Balkan RABV isolates (*N = *210) were found to possess genetic structure (with five genetic groupings), whose relationships better corresponded to geographic region than to time (McElhinney et al., 2011). In contrast with the more rapid evolution detected here for VHSV‐IVb, McElhinney et al. (2011) found that the genetic composition of RABV did not vary from samples collected 20 years earlier. As in VHSV‐IVb, genetic similarity among isolates of another rhabdovirus—Vesicular Stomatitis Virus (VSV) from cattle outbreaks in Mexico—supported their greater relationship to geographic location than to time periods (Velazquez‐Salinas et al., 2014). We discerned that spatial structure of VHSV‐IVb exhibited more pronounced patterning than did temporal structure, although both were significant.

Viruses that are more evolutionarily distant from VHSV also provide spatial and temporal analogies. For example, the *VP* gene of Infectious Bursal Disease Virus (IBDV) displayed more pronounced variation differences among regions in the Iberian Peninsula (24%), than across a 20‐year time span (3%; Cortey, Bertran, Toskano, Majó, & Dolz, 2012). Ebola Virus (EBOV) outbreaks from 2013 to 2015 showed more genome changes earlier than later (Hoenen et al., 2015). However, Gire et al.'s (2014) examination of a longer‐term relationship of EBOV isolates from the 2014 Western African outbreak uncovered significant genetic divergence from older 1970s' outbreaks. These cumulative investigations indicate considerable variation among different viruses in whether, and how much, genetic diversification occurs over time and in whether geographic population genetic structure develops, reflecting complex coevolutionary patterns with their hosts.

### 4.3 Substitution rates, types, and patterns

VHSV‐IVb appears to have evolved at a fairly consistent rate over time, from 2005 to 2017 in the Great Lakes' region. We previously determined its partial *G*‐gene region rate to be 2.8 × 10^−4^ substitutions/nucleotide site per year (Stepien et al., 2015), which was slightly faster than our 1.0 × 10^−4^ estimate here, based on 77 additional isolates (based on the sequences we used for the present population genetic analyses). The calculated rate for the entire *G*‐gene here, which added more conserved (slowly evolving) regions, is 8.5 × 10^−5^ (Table 3). The rate based on our sequences across the entire genome is 6.6 × 10^−5^ (Table 3), which matches the partial gene rate calculation (from a combination of partial *G‐, N‐, Nv‐, M‐*, and *P*‐gene sequences) of 6.6 × 10^−5^ calculated in Stepien et al. (2015).

Nonsynonymous substitutions constituted 41% of the 34 substitutions in the partial *G*‐gene region examined here (668 NT). For the entire *G‐*gene (1,523 NT), this was 48.6% of 37 substitutions found for 44 isolates. Such diversification may enhance the virus' ability to infect new hosts. We discerned 97.5% sequence similarity and 96.4% AA similarity among the *G*‐gene haplotypes, versus 97.7% and 97.5% with the full‐genome sequences (Table 3). Most *G*‐gene haplotypes differed by just single substitutions (62%). In comparison, using a shorter 360 NT *G*‐gene region, Benmansour et al. (1997) identified 92% AA conservation between the Northeastern Pacific VHSV‐IVa subgenogroup (six isolates) and the European VHSV genogroups I–III (eight isolates), and 98% similarity within IVa. Stone, Way, and Dixon (1997) found comparable results across 741 NTs, with IVa (10 isolates) and European genogroups (19 isolates) being 82% similar, and 96% within IVa. The overall number of AA changes and evolutionary rate of IVb to date (i.e., 2003–2017) likely reflects its generalist host range and/or lack of coevolutionary time (and host defense development) in the Great Lakes, since its first outbreaks in 2005.

In comparison, IHNV displayed 91% sequence conservation for a 303 NT *G*‐gene segment of 323 Northeastern Pacific coastal isolates (Kurath et al., 2003). Complete IHNV *G*‐gene sequences showed 94.4% sequence conservation among 38 international samples (Nishizawa, Kinoshita, Kim, Higashi, & Yoshimizu, 2006). *G*‐gene sequences from 61 rabies (RABV) cases in Brazilian livestock had 98% NT similarity and 97% AA conservation, with 18/27 (67%) SNPs resulting in AA changes (Cargnelutti et al., 2017). A comparable study in India examined 25 full‐length *G*‐gene RABV sequences from six host species, revealing 96% sequence and 96% AA conservation (Cherian et al., 2015). Those AA changes occurred away from antigenic sites, suggesting conservation of critical gene functions and avoidance of the host immune system (Cherian et al., 2015). Stone et al. (1997) examined VHSV‐I and VHSV‐IVb isolates for an IHNV antigenic site between AA residues 230–231, but found none in VHSV. Our IVb isolates also were conserved at those positions.

Analyses of whole‐genome VHSV‐IVb sequences documented changes across the 11,158 NT genomes of 44 isolates, finding that 16.6% of the 253 SNPs (involving 2.8% of the nucleotides) encoded nonsynonymous changes. In comparison, full‐genome sequencing by Getchell et al. (2017) of three IVb isolates (including a round goby (haplotype “b”) and the gizzard shad isolates designated here as haplotypes “bc” and “bd,” plus the original haplotype “a”) revealed 87 SNPs, with 26 (30%) encoding AA changes. The number of SNPs they detected was about 1/3 of ours, and they found about 2× more AA changes, with us sequencing 11× more isolates (we also reanalyzed their sequences here). Their higher proportion of AA changes thus may be a sample size artifact. A more recent analysis of 11 other VHSV‐IVb whole‐genome positives, including five from the 2017 Cayuga Lake outbreak and four from a 2017 round goby kill at Long Point State Park, NY by Getchell et al. (2019), discerned 379 SNPs, with 123 being nonsynonymous (33.4%), which also is higher than ours for both and might again reflect sampling size or a mutation rate difference. Similar to our results, a Zika Virus study found 16% nonsynonymous changes among 1,030 SNPs across 110 whole genomes (Metsky et al., 2017), close to the proportion we identified here in the partial *G*‐gene sequences and across the entire VHSV‐IVb genome. Expressed genetic variation in VHSV‐IVb thus appears high.

### 4.4 Gene‐specific variation

The *G‐*gene encodes the glycoprotein of the viral capsid, which enables the viral particle to attach and enter the host cell via endocytosis (Kurath, 2012). In response, the fish hosts mount immune defenses against glycoprotein (Dietzgen & Kuzmin, 2012) whose relatively high mutation rate and genetic diversification may help the viral population to avoid detection (Stepien et al., 2015, 2020). Adaptive radiation in the VHSV‐Ia *G*‐gene occurred in European freshwater rainbow trout (*Oncorhynchus mykiss*) culture following flooding of fish farms with marine water that carried marine host‐based VHSV‐Ia into the area (Schönherz, Forsberg, Guldbrandtsen, Buitenhuis, & Einer‐Jensen, 2018). Here, evolutionary diversification of VHSV‐IVb *G*‐gene variants likewise may facilitate localized adaptation to match cell receptors of particular hosts, such as round goby and gizzard shad, in similar manner to that of Ia in rainbow trout.

Of the individual genes we examined, the novirhabdovirus genus‐specific *Nv‐*gene of IVb had more substitutions (3.8%), as previously was observed across all global VHSV genogroups (Pierce & Stepien, 2012), within the European genogroups (Basurco et al., 1995), and for IHNV (He, Ding, He, Yan, & Teng, 2013; He, Yan, Liang, Sun, & Teng, 2014). The evolutionary rate of the *Nv‐*gene (9.76 × 10^−5^) was the highest among the IVb genes (Table 3). Conversely, Getchell et al. (2017) found a single *Nv* substitution in just one of their four IVb genomes, whereas Basurco et al. (1995) identified one synonymous change in one of nine IVa isolates, likely due to the smaller sample sizes of both studies. The *Nv*‐gene encodes a small nonstructural protein (Ammayappan, Kurath, Thompson, & Vakharia, 2011; Biacchesi, 2011) and has the fastest mutation rate among the VHSV genes (Stepien et al., 2015), suggesting its potential role in evading host detection and/or defense (Pierce & Stepien, 2012). Ammayappan and Vakharia (2011) discovered that *Nv*‐ knockout mutants induced cell apoptosis earlier than did nonaltered (wild‐type) VHSV‐IVb and IHNV, implying that *Nv* prolongs the length of infection, resulting in increased transmission potential.

The *M*‐ and *P*‐genes encode proteins that are directly involved in viral replication. The *M*‐protein inhibits host cell immune responses and promotes viral replication (Biacchesi, Béarzotti, Bouguyon, & Brémont, 2002; Ke et al., 2017; Pore, 2012). The *M*‐gene sequence of VHSV‐IVb's haplotype “a” varies by four AAs from that of VHSV‐IVa, with cell culture experiments showing that IVb better inhibited host cell immune responses, whereas IVa was more effective at blocking host cell transcription (Ke et al., 2017). Similar to the function of the *M‐*gene protein, the *P*‐gene's protein inhibits interferon activation and enhances viral replication (Biacchesi et al., 2002; Pore, 2012). *P‐* serves as a chaperone for *N‐*protein synthesis, preventing it from binding to cellular RNA products, and also forms the RNA polymerase complex with the *L*‐protein (Dietzgen & Kuzmin, 2012).

We found that the VHSV‐IVb *M‐* and *P*‐genes possessed fewer substitutions (2.8% and 1.9%, respectively), whose lower diversity indicates that they are more conserved. This likely is due to the importance of the *M*‐ and *P*‐proteins in viral replication. The *M‐*gene contained the greatest proportion of nonsynonymous changes (53%), suggesting selection.

Moderate variation was discerned in the complete coding region of the *N‐*gene (2.3%; 1,215 NT), which had 36% nonsynonymous substitutions. In comparison, partial *N*‐gene sequences (422 NT) from 16 isolates of VHSV genogroups I–IV (including IVa, but not IVb) varied by 2%–16% (Einer‐Jensen, Ahrens, & Lorenzen, 2005). Four IVb genomes sequenced by Getchell et al. (2017) contained seven *N*‐gene synonymous substitutions. Our sample size was ~11× greater than Getchell et al.'s (2017); that is, the lack of nonsynonymous substitutions in their study likely was due to their sample size. The *N*‐gene encodes the nucleocapsid protein involved with balancing viral transcription and replication. *N‐*protein binds to viral RNA, forming a complex that serves as the template for transcription and replication, and additionally interacts directly with *P*‐ and *L‐*proteins at multiple binding sites (Dietzgen & Kuzmin, 2012); thus, its conservation is expected.

The remaining gene, *L* (5,955 NT), has been relatively little studied in VHSV until now. We found that it has evolved the most slowly (1.9%), with 33.9% of its mutations being nonsynonymous. Schönherz et al. (2016) sequenced the full genomes of four VHSV‐Ia isolates, finding the most mutations in the *G‐* and *L*‐genes. The latter is true here, due to the length of the *L*‐gene, rather than its rate. The *L*‐protein functions in viral transcription and replication (Kurath, 2012), with its variants increasing the virus' temperature range to >20°C, as demonstrated with gene‐swapping experiments (Kim, Yusuff, Vakharia, & Evensen, 2015).

### 4.5 Host species generality, specificity, and infection

Less than 1% of the individual fishes from our 2015 to 2017 sampling tested VHSV positive. In comparison, 2010 samples from the Great Lakes described 13% VHSV‐IVb incidence among >5,000 fish individuals, with 25% of those occurrences found in round goby, which was targeted due to its high infection incidence (Cornwell et al., 2015). That overall incidence was a decrease from 16% positive occurrences found in 2009 (Cornwell, Eckerlin, et al., 2012). Our results indicate that VHSV infection has continued to decrease, with no cases reported from 2018 to 2020. More similar to our results, 2009–2011 sampling in coastal Norway identified <1% of 943 fishes as VHSV‐I positive (Sandlund et al., 2014). Moreover, sampling for VHSV‐Id around Finland in 2005–2008 detected no positives in wild fishes (1,636 individuals, 17 species) despite collection near aquaculture net pens that had experienced recent outbreaks (Vennerström et al., 2018). Thus, global VHSV occurrences and predictability appear sporadic.

All dreissenid mussels tested by us were VHSV negative. Throckmorton et al. (2017) likewise discovered no VHSV in cylindrical papershell mussels (*Anodontoides ferussacianus*) from Budd Lake, despite positive largemouth bass and Hyalellidae amphipods from that location. Researchers in Denmark tested for a VHSV‐II invertebrate reservoir in isopods, krill, and squid, but none were positive (Skall, Olesen, & Mellergaard, 2005). To better understand whether invertebrates may serve as a reservoir or transmission vector, comprehensive sampling and testing during VHSV outbreaks should be conducted, along with challenge studies on specific taxa, focusing on species in areas experiencing outbreaks.

Across all VHSV genogroups I–IV worldwide, an average of three additional fish species each year since 1962 have been identified as new hosts (Escobar et al., 2018). Among VHSV genogroups, IVb has infected the broadest host range, suggesting greater ability to spread to naïve species (Escobar et al., 2018), which appears to be the case for its evolutionary patterns described here. A wide variety of host taxa often characterizes a new, unspecialized virus (Kitchen, Shackelton, & Holmes, 2011), as fits VHSV‐IVb. Our research discerned little support for trends in infected host species and specialization among IVb haplotype groupings to date. Similar to our geographic findings, VHSV‐IVa isolates from eight fish species showed higher *G*‐gene similarity to isolates from the same geographic region than to those from the host species (Hedrick et al., 2003). Moreover, no host species associations were found for 63 full *G*‐gene IVa isolates collected over a 20‐year span, with just four of those haplotypes recovered from the same host species (Garver et al., 2013).

Much differentiation of the VHSV‐IVb haplotype “b” group occurred in round goby from the Lower Great Lakes region during the Middle and Later time periods, suggesting that this invasive species might serve as a reservoir or vector (also see Cornwell et al., 2014). Many round goby individuals collected during and outside of large VHSV‐IVb outbreaks displayed classic hemorrhaging and other signs of disease (Cornwell, Getchell, Groocock, Walsh, & Bowser, 2012; Groocock et al., 2007), yet others had none (this study and Cornwell, Eckerlin, et al., 2012). Round goby has become one of the most common benthic fishes in the lower Great Lakes (Snyder & Stepien, 2017) and is a major prey item for game fishes (Johnson, Bunnell, & Knight, 2005), possibly aiding VHSV transmission among species. Two round goby individuals from the peak of a small outbreak each possessed two different IVb haplotypes (Cornwell et al., 2014); such variation within individuals could increase transmission likelihood, since the host might fail to recognize some newer sequence variants. Getchell et al. (2019) also reported polymorphisms at single NT positions in some isolates from the Cayuga Lake 2017 outbreak. Here, we found no evidence of multiple haplotypes within a single individual. Coinfections by multiple isolates are not easy to detect, as the sequence having more RNA will exhibit greater PCR amplification (Hoorfar et al., 2004).

The round goby's large population sizes and relatively high genetic diversity levels (Brown & Stepien, 2009; Snyder & Stepien, 2017) may facilitate VHSV‐IVb's coevolutionary success. The goby's high VHSV‐IVb infection incidence may suggest that the virus is in the process of evolving some host specificity (Cornwell et al., 2014), notably in the “b” haplotype group indicated here. Since past research targeting round goby mostly focused on Lake Ontario (Cornwell et al., 2014) and the St. Lawrence River (Groocock et al., 2007), future efforts also should examine other populations.

Gizzard shad was the second most commonly infected species (22 VHSV‐IVb isolates), dominating the original 2006 Lake Erie outbreak (primarily haplotype “a”; Thompson et al., 2011; C. A. Stepien, personal observation, 2006). The 2017 Lake St. Clair outbreak also primarily infected gizzard shad (M. Faisal & G. Whelan, personal communications, 2017), comprising a single unique haplotype (“be”). Many gizzard shad positives occurred during the Later time period in the Central Great Lakes, suggesting a possible reservoir species. Gizzard shad have a high death rate from VHSV (Thompson et al., 2011). We also here report the first IVb detection in alewife (Lake Michigan, 2016), which, like gizzard shad, belongs to the Clupeid family.

Unlike VHSV, IHNV has a much narrower host range, exclusively infecting certain salmonid species in the Northeastern Pacific (Lapatra, 1998). Within its “U” group, subgroup “UP” has been more common in sockeye salmon (*Oncorhynchus nerka*), whereas “UC” infected Chinook salmon (*O. tshawytscha*) and steelhead trout (*O. mykiss*), reveal some geographic regional differences (Black et al., 2016). Similarly, group “b”‐derived haplotypes of VHSV‐IVb might develop more specificity in round goby over time, meriting future investigation.

In comparison, high RABV genetic diversity is believed to have facilitated jumps among a wide taxonomic range of key host species, including bats, raccoons, and many canids (Rodríguez‐Nevado, Lam, Holmes, & Pagán, 2018). A genetically diverse host pool, whether in terms of population or species, can increase diversity of viral sequences (Ojosnegros & Beerenwinkel, 2010); this appears to be the case for VHSV‐IVb.

The effects of VHSV‐IVb on different host species, as well as within a given host species, varied considerably in our study, as measured by concentrations of the virus in infected field‐collected fish tissues determined with our qPCR assay. Our qPCR assay used internal standards (IS), increasing measurement accuracy (Pierce, Willey, Crawford, et al., 2013; Pierce, Willey, Palsule, et al., 2013). Similar breadth in virus titers characterized individual fish in viral challenge experiments infected with haplotype “a” (Pierce, Willey, Crawford, et al., 2013; Pierce, Willey, Palsule, et al., 2013). Notably, four of our field‐collected fish samples were above the threshold for clinical onset of disease signs in haplotype “a” laboratory‐challenged fish (Pierce, Willey, Palsule, et al., 2013). The 2015 round goby positive possessed the highest recorded level of VHSV‐IVb, yet had no physical signs of infection. Concentrations in three gizzard shad individuals also were above the clinical threshold, despite appearing free of disease. Virus concentrations in the majority (67%) of the 2015–2016 isolates we tested were below the cell culture detection threshold used in other studies, demonstrating that the use of IS in the qPCR assays developed by Pierce, Willey, Crawford, et al. (2013), and by Pierce, Willey, Palsule, et al. (2013) shows high ability to detect VHSV in host species. The virus' titers, as well as its effects on the immune system of the fish hosts, appear to vary considerably among haplotypes, individuals, and species. This merits further investigation with challenge experiments.

### 4.6 Selection and coevolution

Tajima's *D* analysis results indicate that the *G*‐gene and the VHSV‐IVb genome as a whole, have evolved under significant purifying selection. We found evidence of purifying selection acting on single *N‐* and *G‐*gene codons, and on six codons in the *L‐*gene, based on results of Bayesian analyses in FUBAR and MEME. Other studies have found that purifying selection regulated VHSV evolution in other genogroups (Abbadi et al., 2016; He et al., 2014), as well as in other rhabdoviruses (Kuzmin, Novella, Dietzgen, Padhi, & Rupprecht, 2009) and RNA viruses in general (Hughes & Hughes, 2007).

Diversifying selection was indicated for two VHSV‐IVb genes: *G* and *Nv,* in our results. Positive selection was implicated for changes in two IVb *G*‐gene codons. Positive selection, as well as diversifying selection, on viral genes constitute support for the host–pathogen “arms race,” during which the virus's evolutionary course acts to thwart and/or suppress host immune responses (Pereira & Amorim, 2013).

Following a quasispecies pattern, VHSV‐IVb radiated into many closely related variants (toward the possible result of evading recognition by the host's immune system), with purifying selection removing less fit ones from the viral gene pool. The original haplotypes “a” and “b” disappeared over time, likely resulting from the increasing recognition of the virus by the fish hosts' immune systems and the growing resistance of the host populations. This increasing genetic diversification of the virus presumably enabled some newer and rarer VHSV variants to elude the hosts' immune system recognition and delay their antiviral responses. Genetic differentiation in descendent virus populations allowed the virus to persist over time, albeit at lower levels and with less virulence.

Like VHSV‐IVb, Spring Viremia of Carp Virus (SVCV) and IHNV populations underwent purifying selection (He et al., 2013; Padhi & Verghese, 2012). Purifying selection pressures on IHNV appeared greater than for VHSV‐IVb, attributed to the former's more limited reported realm of host species and its longer temporal history (He et al., 2013). Analysis of 27 rhabdovirus taxa revealed that purifying selection has characterized all members and was greatest in the *Lyssavirus* genus (including RABV; Kuzmin, Hughes, & Rupprecht, 2006). In the case of RABV in Taiwan, purifying selection in the viral population increased in response to reductions in vaccinating its potential host populations, as infection incidences expanded (Lin et al., 2016). Global RABV evolutionary patterns have displayed strong purifying selection across mammalian hosts on their overall phylogenetic tree, being most pronounced in the bat and dog clades that are some of that virus' most prevalent hosts (Troupin et al., 2016). It appears likely that VHSV evolutionary patterns will continue to be shaped by purifying selection.

Illustrating local host population resistance, Millard, Brenden, LaPatra, Marcquenski, and Faisal (2014) discerned that VHSV‐IVb antibodies lasted up to one year in muskellunge. Throckmorton, Peters, Brenden, and Faisal (2015) detected VHSV‐IVb from Budd Lake in 2011 following its absence since 2007, suggesting an interlude of dormancy due to local immunity. Similar to our findings, a study of Danish VHSV‐Ia isolates distinguished an almost four‐year gap between fish kills, despite some detections in local fishes (Kahns et al., 2012). This may explain the relative rarity of VHSV‐IVb from 2010 to present in Great Lakes populations. However, our results show that during these interludes, the virus continued to evolve to maintain “a foothold” in the local populations. VHSV‐IVb thus underwent considerable diversification over time and space, whose trajectory may continue.

## 5 SUMMARY AND CONCLUSIONS

VHSV‐IVb appears to have remained geographically constrained to the Great Lakes region and surrounding areas, albeit at lower incidence and virulence over time, and continued to mutate and genetically diversify between outbreaks. Significant sequence diversification of its viral populations occurred both spatially and temporally. This coevolutionary pattern likely aided the virus in circumventing immune system recognition by the hosts, in response to the increasing resistance of the host populations. More recently, the virus has displayed reduced virulence, as well as evolution toward possible persistence at low levels in resident host populations. It also is likely that VHSV‐IVb may continue to infect naïve fish populations and could spread to other water bodies, facilitated by its quasispecies pattern of continuing evolutionary differentiation.

## CONFLICT OF INTEREST

None declared.

## AUTHOR CONTRIBUTION

**Carol A. Stepien:** Conceptualization (lead); Data curation (equal); Formal analysis (equal); Funding acquisition (lead); Investigation (equal); Methodology (equal); Project administration (lead); Supervision (lead); Validation (equal); Visualization (equal); Writing‐original draft (equal); Writing‐review & editing (lead). **Megan D Niner:** Data curation (equal); Formal analysis (equal); Investigation (equal); Methodology (equal); Validation (equal); Visualization (equal); Writing‐original draft (equal); Writing‐review & editing (supporting).

## Data Availability

DNA sequences are deposited and reported in GenBank (https://www.ncbi.nlm.nih.gov/genbank/), with accession numbers provided in Tables 1 and 2. Data files and analyses are deposited and reported in Dryad (https://doi.org/10.5061/dryad.8cz8w9gmh).
